# The Specific NLRP3 Antagonist IFM-514 Decreases Fibrosis and Inflammation in Experimental Murine Non-Alcoholic Steatohepatitis

**DOI:** 10.3389/fmolb.2021.715765

**Published:** 2021-08-13

**Authors:** Sandra Torres, Maximilian J Brol, Fernando Magdaleno, Robert Schierwagen, Frank E. Uschner, Sabine Klein, Cristina Ortiz, Olaf Tyc, Nadine Bachtler, James Stunden, Damien Bertheloot, Ana Kitanovic, Brian Sanchez, Jacob Schrum, William R. Roush, Luigi Franchi, Kate Byth, Eicke Latz, Jonel Trebicka

**Affiliations:** ^1^ Translational Hepatology, Department of Internal Medicine I, Universitätsklinikum/ Goethe-Universität, Frankfurt, Germany; ^2^ Department of Internal Medicine I, University Clinic Bonn, Bonn, Germany; ^3^ IFM Therapeutics, Boston, MA, United States; ^4^ Institute of Innate Immunity, University Clinic Bonn, Bonn, Germany; ^5^ IFM Therapeutics, Ann Arbor, MI, United States; ^6^ European Foundation for the Study of Chronic Liver Failure – EF Clif, Barcelona, Spain

**Keywords:** inflammasome, liver fibrosis, steatosis, NASH, caspase-1

## Abstract

**Background and Aims:** Activation of the inflammasome NLRP3 (NOD-, LRR- and pyrin domain containing 3) contributes to the development of non-alcoholic fatty liver disease (NAFLD) and progression to non-alcoholic steatohepatitis (NASH). Therefore, this study explored the therapeutic effects of a novel and selective NLRP3 antagonist in a murine dietary model of NASH.

**Methods:** Groups of 12-week-old *ApoE* ^-/-^ mice were fed ad lib for 7 weeks with a methionine/choline deficient (MCD) and western diet (WD). After 3 weeks of diet-induced injury, mice were injected i. p. with the NLRP3 antagonist IFM-514 (100 mg/kg body weight) or vehicle (0.5% carmellose) every day, 5 days/week for a further 4 weeks. Several markers of inflammation, fibrosis and steatosis were evaluated. Whole transcriptome sequencing and panel RNA expression analysis (NanoString) were performed.

**Results:** IFM-514 inhibited IL-1*β* production in mice challenged with 20 mg/kg lipopolysaccharide, and in mouse and human inflammatory cells *in vitro*. IFM-514 inhibited hepatic inflammation in the *in vivo* non-alcoholic steatohepatitis model assessed by H&E staining and in the hepatic gene expression of inflammasome-related proinflammatory cytokines. This effect was associated with significant reduction in caspase-1 activation. Similarly, IFM-514 was efficacious *in vivo* in MDC-fed *ApoE* ^-/-^ mice, markedly reducing portal pressure, Sirius red staining and 4-hydroxyproline content compared to vehicle-treated mice. Moreover, IFM-514 significantly reduced hepatic steatosis in MCD-fed *ApoE* ^-/-^ mice, as evidenced by NAFLD scores, oil red O staining, hepatic triglycerides and gene expression. In WD treated animals, similar trends in inflammation and fibrosis were observed, although not sufficient IFM-514 levels were reached.

**Conclusion:** Overall, IFM-514 reduced liver inflammation and fibrosis, with mild effects on liver steatosis in experimental murine NASH. Blocking of NLRP3 may be an attractive therapeutic approach for NASH patients.

**GRAPHICAL ABSTRACT:**
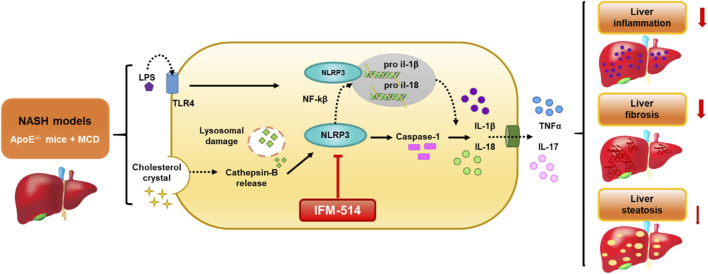
Schematic representation of the IFM-514 treatment effect in experimental murine NASH. The activation of NLRP3 inflammasome in response to NASH models is due to the gut-derived PAMPs, such as LPS, and cholesterol crystals. LPS activates nuclear factor kappa B (Nf-kB) via triggering of the TLR4 receptor and consequently promotes the expression of pro-IL-1β and pro-IL-18. Cholesterol crystals produce lysosomal damage with cathepsin-B release that also activates NLRP3, followed by caspase-1 activation that allows the release of IL-1β and IL-18 into the extracellular space and increasing TNF-α and IL-17. All these cytokines promote an increase in liver inflammation, fibrosis and steatosis. In our study, IFM-514, an NRLP3 antagonist, could protect prior to the pathogenesis produced by NASH murine model, with mild effect in liver steatosis.

## Highlights

⁃ IFM-514 reduced liver inflammation and fibrosis in MCD-fed *ApoE* ^-/-^ mice.
⁃ IFM-514 produced a mild reduction in liver steatosis in MCD-fed *ApoE* ^-/-^ mice.
⁃ Blocking NLRP3 might be an attractive therapeutic approach for NASH patients.

## Lay Summary

The activation of NLRP3 inflammasome in response to NASH models is due to the gut-derived PAMPs, such as LPS, and cholesterol crystals. LPS activates nuclear factor kappa B (Nf-kB) *via* triggering of the TLR4 receptor and consequently promotes the expression of pro-IL-1*β* and pro-IL-18. Cholesterol crystals produce lysosomal damage with cathepsin-B release that also activates NLRP3, followed by caspase-1 activation that allows the release of IL-1*β* and IL-18 into the extracellular space and increasing TNF-*α* and IL-17. All these cytokines promote an increase in liver inflammation, fibrosis and steatosis. In our study, IFM-514, an NRLP3 antagonist, could protect prior to the pathogenesis produced by NASH murine model, with mild effect in liver steatosis.

## Introduction

Non-alcoholic fatty liver disease (NAFLD), characterized by fat accumulation in the liver (steatosis) in the absence of chronic alcohol use, is a common and emerging cause of chronic liver disease (Williams et al., 2011; Rinella, 2015). Interestingly, NAFLD is present in obese as well as lean patients. However, little is known about the differences between these two NAFLD types and thus, no tailored therapeutic approaches are available. Moreover, an estimated 10–15% of NAFLD patients develop non-alcoholic steatohepatitis (NASH), while a quarter of those will develop liver cirrhosis and potentially hepatocellular carcinoma (Macaluso et al., 2015). Therefore, NASH represents the severe and dangerous form of NAFLD characterized by hepatocyte injury, inflammation and fibrosis - the most critical outcome in NASH - , that may result from inflammasome activation (Ganz et al., 2015).

Inflammasomes are cytoplasmic multiprotein complexes that can sense danger signals from damaged cells and pathogens. They assemble to mediate caspase-1 activity, secretion of cytokines and other pro-inflammatory mediators, including IL-1*β* and IL-18 (Martinon et al., 2002; Schroder and Tschopp, 2010; Szabo and Petrasek, 2015), as a result of tissue damage or cellular stress. Several members of the NLR family (nucleotide-binding and oligomerization domain and leucine-rich-repeat-containing proteins), including the NOD-like receptor protein 3 (NLRP3), have been linked to the pathophysiology of NASH (Abstract diagram). This has inspired strategies to block inflammasome activation by pharmacological targeting of NLRP3 (Próchnicki et al., 2016). In fact, inhibition of NLRP3 can be achieved by limiting Toll-like receptor- and tumor necrosis factor-mediated increases in NLRP3 expression. However, since this approach lacks specificity and is likely to produce many off-target effects (Bahia et al., 2015), this study tested a novel selective NLRP3 antagonist for therapeutic effects in murine models of NASH. Since both NASH and NLRP3 have been linked to metabolic syndrome we have chosen murine models for which we have previously demonstrated that it is suitable for investigation of liver of metabolic syndrome (Schierwagen et al., 2015, 2016).

## Results

### IFM-514 Inhibits the NLRP3 Inflammasome *In Vivo* and *In Vitro*

**FIGURE 1:**
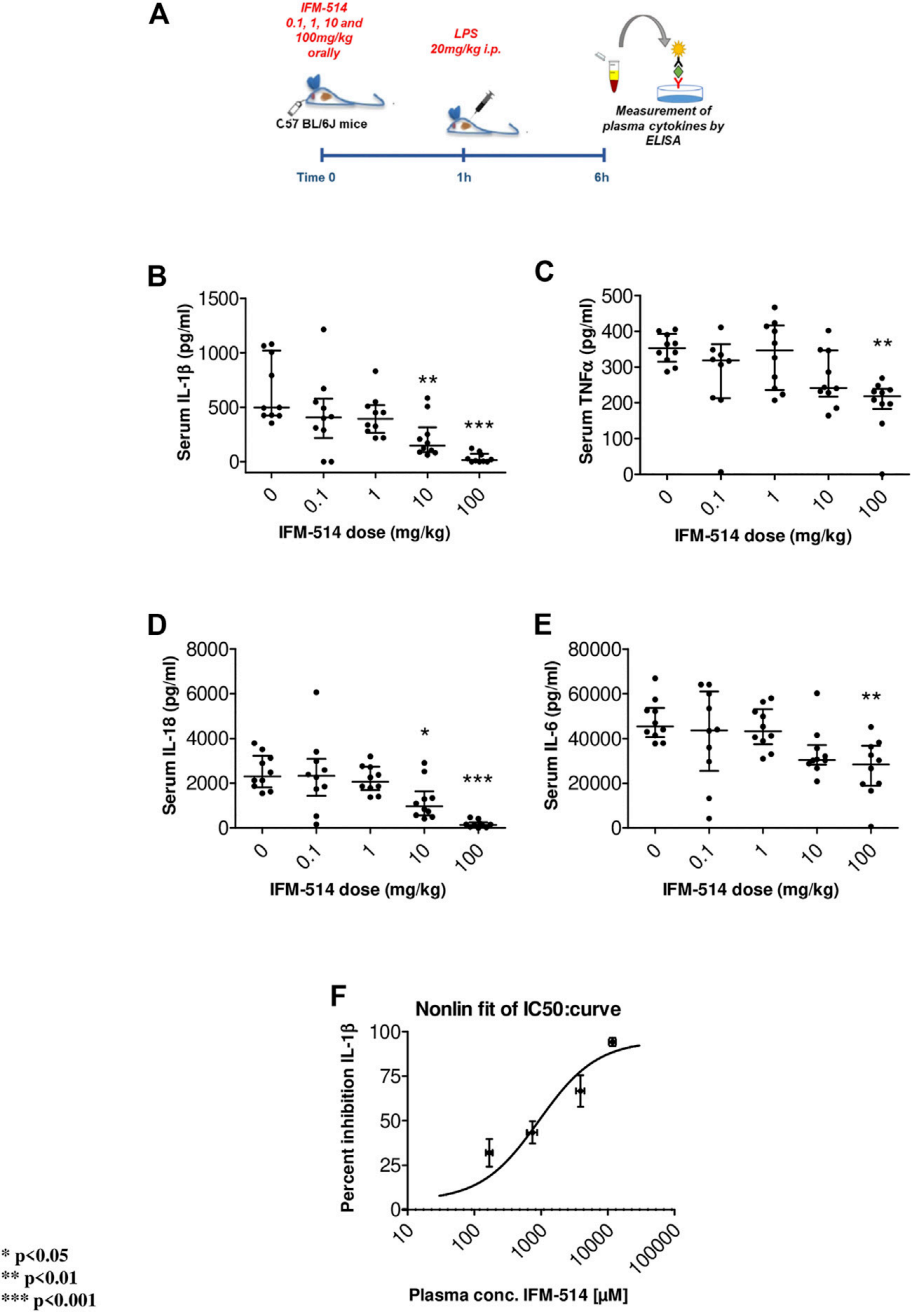
Experimental design for mouse *in vivo* pharmacodynamics model: LPS stimulation of plasma cytokines. IFM-514 was dosed orally in WT C57BL/6 mice at 0.1, 1, 10 and 100 mg/kg 1 h before LPS (20 mg/kg i. p.) **(A)**. LPS-induced production of plasma cytokines IL-1*β* **(B)**, TNF*α* **(C),** IL-18 **(D)** and IL-6 **(E)** measured by ELISA 5 h after LPS challenge. IFM-514 significantly inhibited IL-1*β* production *in vivo* when dosed orally with an IC50 = 947 ng/ml; 95% CI 499.5–1,683 ng/ml **(F).** Results are expressed as mean ± standard error of the mean (SEM); n = 10/group, *p < 0.05, **p < 0.01 and ***p < 0.001. Abbreviations: ELISA, enzyme-linked immunosorbent assay; IC50, half maximal inhibitory concentration; IL1*β*, interleukin 1*β*; IL-16, interleukin 16; IL18, interleukin 18; LPS, lipopolysaccharide; TNF*α*, tumor necrosis factor *α*.

In human cells, IFM-514 inhibited gramicidin-induced IL-1*β* release in PMA-primed human THP-1 cells (half maximal inhibitory concentration, IC_50_ 0.275 ± 0.115 µM, n = 9) and was a potent inhibitor of gramicidin-induced IL-1*β* release in LPS-primed monocyte-derived macrophages (IC_50_ 0.156 ± 0.042 µM, n = 8). Similarly, IFM-514 was a potent inhibitor of gramicidin-induced IL-1*β* release in LPS-primed immortalized macrophages from WT C57BL/6 mice (IC_50_ 0.146 ± 0.042 µM, n = 6), displayed similar potency in primary bone marrow-derived macrophages (BMDM) from C57BL/6 mice (data not shown) and in BMDM from Balb/c mice (IC_50_ 0.313 ± 0.013 µM, n = 6; free IC_50_ 10.9 ± 0.5 nM. To determine the recommended dose *in vivo*, WT C57BL/6 mice were used in a pharmacodynamic (PD) model of lipopolysaccharide (LPS) mediated cytokine release. IFM-514 was dosed orally in mice at 0.1, 1, 10 and 100 mg/kg 1 h before LPS challenge (20 mg/kg i. p.) and plasma cytokines were measured 5 h later by ELISA (Figure 1A). IFM-514 strongly and dose-dependently abrogated the LPS-induced production of NLRP3-dependent plasma cytokines IL-1*β* and IL-18, and had a mild but significant effect on TNF*α* and IL-6 at the highest dose tested (Figures 1B–E). Moreover, the 100 mg/kg dose of IFM-514 inhibited IL-1*β* production *in vivo* by more than 90%, even when dosed orally. The plasma concentration of IFM-514 was measured at the time of cytokine measurement and plotted against the percentage inhibition of IL-1*β* production, resulting in an IC_50_ of 947 ng/ml (95% CI 499.5–1,683 ng/ml) (Figure 1F). IFM-514 was highly protein bound in plasma (99.59% bound), to an extent that, when adjusted for plasma protein binding, the free IC_50_ *in vivo* was 3.9 ng/ml or 9.2 nM, which corresponded with the free *in vitro* IC_50_ in mouse BMDM. Consistently, single-dose pharmacokinetic (PK) studies in WT C57BL/6 mice suggested that the recommended dose for *in vivo* use was 100 mg/kg free base (corresponding to 105 mg/kg Na + salt) when delivered i. p. or orally once-per-day (Supplementary Table 1). Using modeled PK data, this dose gave an average free liver concentration above the free *in vivo* IC_90_ in the LPS challenge model (83 nM) and the free *in vitro* IC_90_ in the BMDM assay (40.0 ± 3.5 nM) for approximately 24 h. Therefore, this dose was used for the *in vivo* experiments in NASH-induced *ApoE* ^-/-^ mice (Table 1).

**TABLE 1:** Age, body weight (week 0 and 7), liver weight and liver-to-body weight ratio of *ApoE* ^*-/-*^ mice receiving normal diet, MCD, WD and IFM-514-treated MCD-fed and WD-fed mice.

Mice group	Age (weeks)	Week 0 body weight (g)	Week 7 body weight (g)	Liver weight (g)	Liver-to-body weight ratio
Normal Diet	19 ± 0.0	25.2 ± 1.20	28.6 ± 1.18	1.4 ± 0.13	4.9 ± 0.38
MCD	21.2 ± 1.40	26.6 ± 3.75	16.7 ± 2.73^b^	0.9 ± 0.15	5.2 ± 0.59
MCD + IFM-514	16.3 ± 1.96	22.3 ± 3.88	14.3 ± 2.27^c^	0.7 ± 0.07	5.4 ± 0.40
WD	12.3 ± 2	23.3 ± 2.90	24.3 ± 3.86^b^	1.3 ± 0.25	5.5 ± 0.45
WD + IFM-514	13 ± 2.13	21.8 ± 3.83	24.0 ± 3.62^d^	1.3 ± 0.26	5.8 ± 2.25

a No significant differences in body weight between any groups in week 0.

b versus control diet.

c versus MCD.

d versus WD.

**FIGURE 2:**
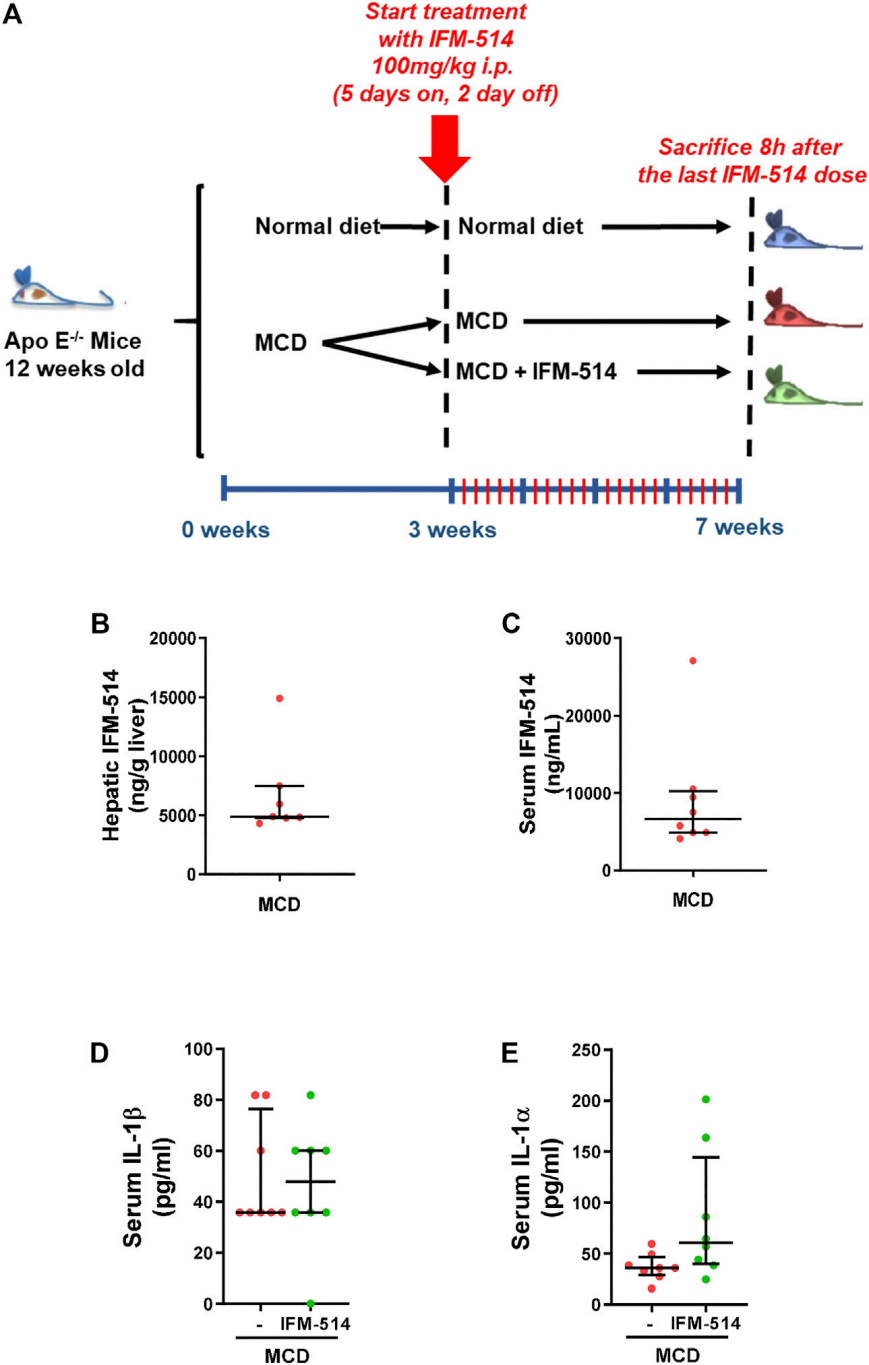
Study design and interleukin levels. Groups (n = 10) of 12-week-old *ApoE* ^*-/-*^ mice were fed *ad lib* for 7 weeks with methionine/choline deficient (MCD) diet. After 3 weeks of diet-induced injury, mice were injected i. p. with the NLRP3 antagonist IFM-514 (100 mg/kg body weight) or vehicle (0.5% carmellose) every day, 5 days/week for a further 4 weeks **(A)**. Concentration of IFM-514 in liver (ng/g of tissue) and serum (ng/ml) following i. p. administration of IFM-514 in NASH-induced mice for 20 consecutive days. The concentration was measured by HPLC-MS **(B–C)**. Circulating IL-1*β* and IL-1*α* in IFM-514-treated MCD-fed *ApoE* ^*-/-*^ mice **(D–E)**. Results are expressed as mean ± standard error of the mean (SEM); n = 10/group, *p < 0.05, **p < 0.01 and ***p < 0.001. Abbreviations: IL1*α*, interleukin 1*α*.

Here, 12-week-old *ApoE* ^-/-^ mice were fed for a total of 7 weeks with MCD diet. After 3 weeks of diet-induced injury, mice received i. p. injections of IFM-514 (100 mg/kg) or vehicle for 5 days/week for a further 4 weeks (Figure 2A). To determine whether the liver was adequately exposed to IFM-514, the concentration of IFM-514 was measured by HPLC-MS in liver and serum 8 hours after the last dose of IFM-514 in MCD-fed *ApoE* ^-/-^ mice (Figures 2B,C). The free IFM-514 concentration in the liver was above the free plasma concentration and sufficient to inhibit 90% of the IL-1*β* synthesis in the LPS-PK/PD model. IFM-514-treated NASH *ApoE* ^-/-^ mice had similar circulating IL-1*β* and IL-1*α* as vehicle-treated NASH *ApoE* ^-/-^ mice (Figures 2D,E).

### IFM-514 Reduces Hepatic Inflammation in MCD-Fed *ApoE* ^-/-^ Mice

**FIGURE 3:**
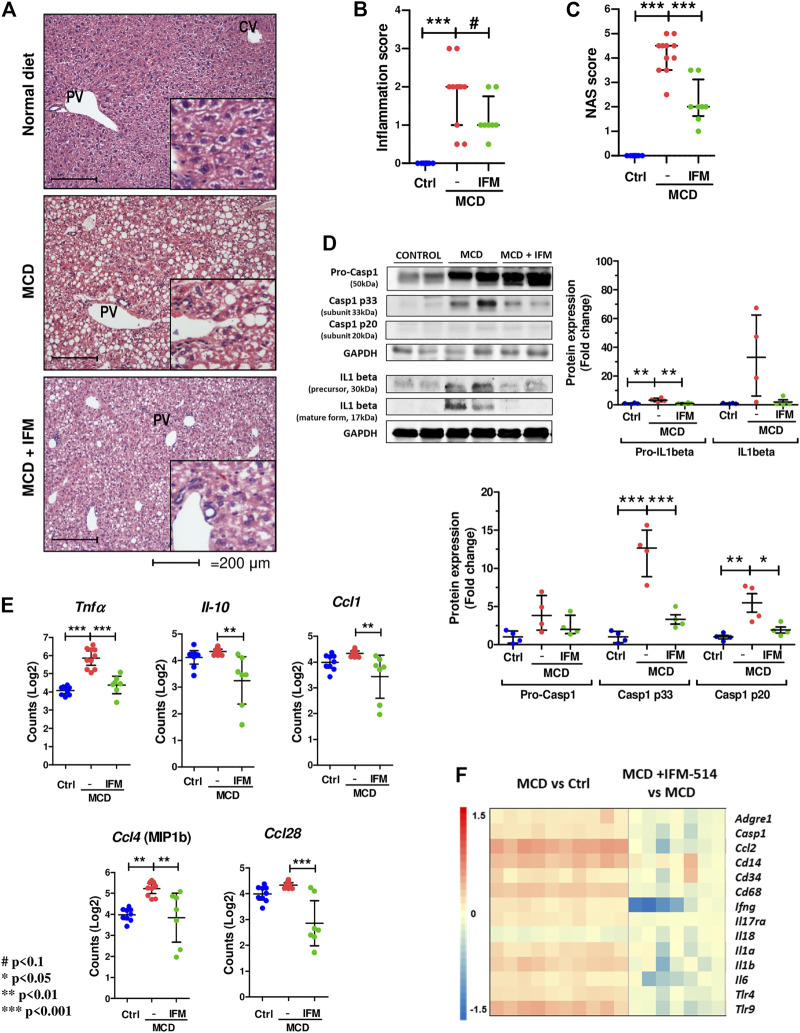
IFM-514 reduces hepatic inflammation in MCD-fed *ApoE* ^*-/-*^ mice. H&E staining **(A)** inflammation score **(B)** and NAS score **(C)** in IFM-514-treated MCD-fed *ApoE* ^*-/-*^ mice. Caspase-1 p33, caspase-1 p20, pro-caspase-1, mature IL-1*β* and pro-IL-1*β* protein expression **(D)**. Inflammasome-related gene expression **(E)**. Heatmap for hepatic inflammation set of genes **(F)**. Scale and genes are provided on heatmap. Results are expressed as the mean ± standard error of the mean (SEM); n = 10/group, ^#^ *p* < 0.1. *p < 0.05, **p < 0.01 and ***p < 0.001. Representative photomicrographs were captured at ×100 (scale bars = 200 μm) and ×200 magnification (scale bars = 100 μm). Abbreviations: Ccl1, chemokine (C-C motif) ligand 1; Ccl4, chemokine (C-C motif) ligand 4; Ccl28, chemokine (C-C motif) ligand 28; CV, central vein; GAPDH, glyceraldehyde-3-phosphate dehydrogenase; H&E, hematoxylin and eosin; *Il-10*, interleukin 10; PV, portal vein. See Supplementary Table 3 for Heatmap genes.

Since NLRP3-mediated inflammation is linked to NASH (Mridha et al., 2017), we sought to test whether IFM-514 has an effect in MCD-fed *ApoE* ^-/-^ mice. Hematoxylin and eosin (H&E) staining (Figure 3A), inflammation score (Figure 3B) and NAS scores (Figure 3C) showed that IFM-514 reduces hepatic inflammation in MCD-fed *ApoE* ^-/-^ mice, being significant the NAS score. Liver samples were further processed to analyze the activation of caspase-1 and IL-1*β* protein expression, a pathway that is extremely important in NASH (Szabo and Petrasek, 2015). Protein analysis showed that the activation of p20 and p33-caspase-1 subunits and pro-IL-1*β* were significantly reduced in IFM-514-treated vs vehicle-treated MCD-fed *ApoE* ^-/-^ mice, but mature IL-1*β* and pro-caspase-1 showed a tendency to decrease with IFM-514-treatment (Figure 3D). To further pinpoint the mechanism of IFM-514 in hepatic inflammation, liver RNA was extracted for NanoString mouse myeloid innate immunity panel of 770 genes. MCD diet induced upregulation of genes at least 1.5-fold, and some of these genes were clearly reduced in IFM-514-treated vs vehicle-treated NASH *ApoE* ^-/-^ mice. This set of genes included several pro-inflammatory genes, such as chemokine ligands (Ccl) 1, 4 and 28, Il10 and Tnf*α* (Figure 3E), also shown in the heatmap (Figure 3F). In agreement with these data, IFM-514 had a pronounced effect on cytokine and inflammasome-related genes in the MCD-fed mice. Moreover, IFM-514 clearly reduced the hepatic expression of many genes related to the cytokine-cytokine receptor interaction (C-C) pathway, which is not limited to inflammasome function, but inflammation in general. These results suggest that IFM-514 inhibits the NLRP3 inflammasome and the subsequent activation of caspase-1 in MCD-fed *ApoE* ^-/-^ mice. The effect of IFM-514 decreasing liver inflammation was supported by immunohistochemistry of hepatic macrophages and activated kuffer cells (F4/80-positive cells) (Supplementary Figure 1), such as the reduction of *Adgre1* expression indicated in the heatmap (Figure 3F).

### IFM-514 Reduces Hepatic Fibrosis in MCD-Fed *ApoE* ^-/-^ Mice

**FIGURE 4:**
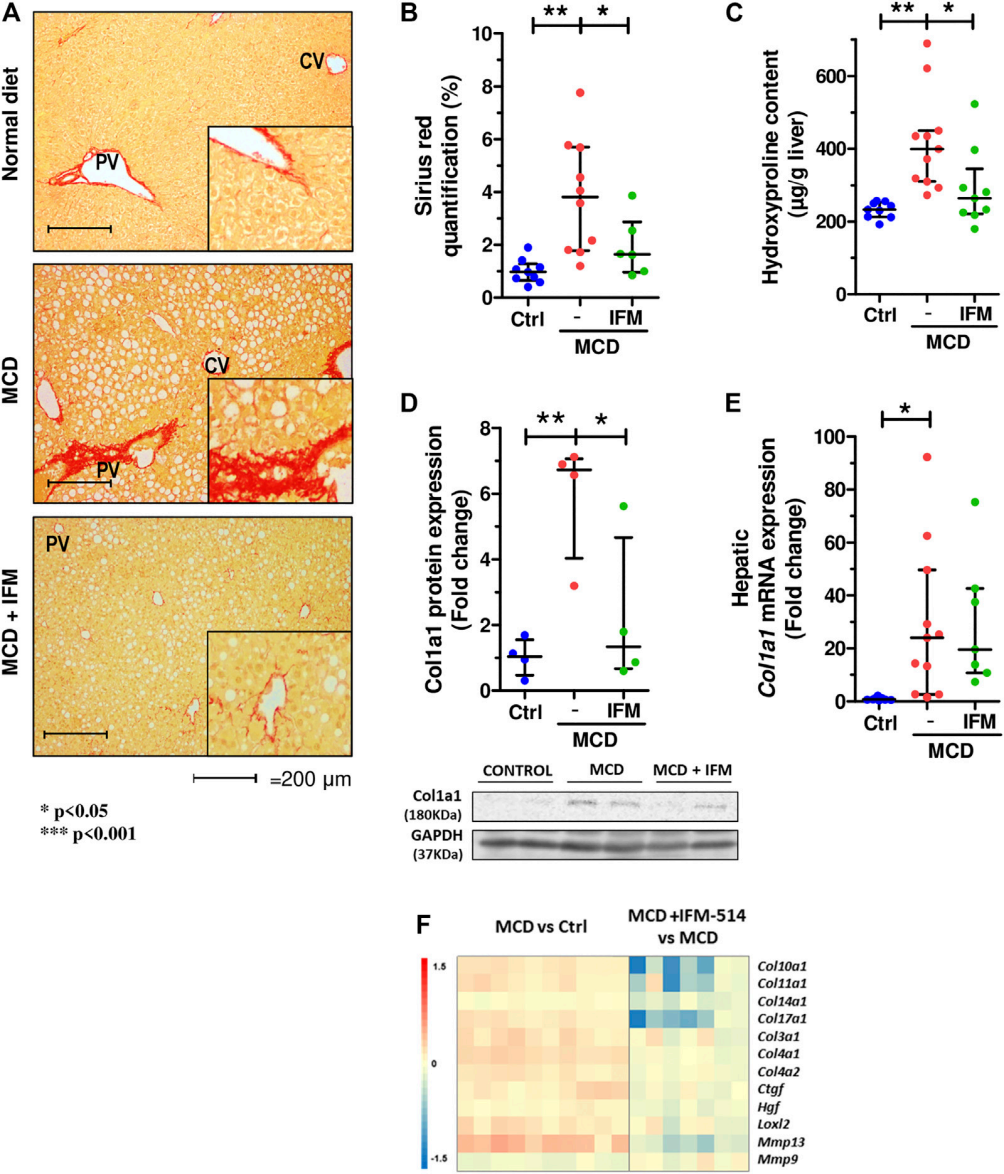
IFM-514 reduces liver fibrosis in MCD-fed *ApoE* ^*-/-*^ mice. Sirius red (SR) staining **(A)** with SR quantification **(B)** and hepatic 4-hydroxyproline content **(C)**. Hepatic Col1a1 protein **(D)** and mRNA expression **(E)** from IFM-514-treated MCD-fed *ApoE* ^*-/-*^ mice. Heatmap for hepatic fibrosis set of genes **(F).** Results are expressed as mean ± standard error of the mean (SEM); n = 10/group, #*p* < 0.1, *p < 0.05, **p < 0.01 and ***p < 0.001. ^$^ *p* < 0.05, if outliers are excluded. Scale bars = 200 μm. Abbreviations: col1a1, collagen type 1 *α*1. See Supplementary Table 3 for Heatmap genes.

Given that MCD feeding induced hepatic fibrosis in *ApoE* ^-/-^ mice, we tested whether the inhibition of NLRP3 with IFM-514 had an effect on markers of fibrosis. IFM-514 treatment significantly reduced liver fibrosis as shown by quantitative Sirius red (SR) staining (Figures 4A,B), and the 4-hydroxyproline content (Figure 4C). Collagen type 1 *α*1 chain (Col1a1) protein expression (Figure 4D) had a significant reduction and hepatic mRNA expression levels (Figure 4E) also had a tendency towards reduction in MCD-fed *ApoE* ^-/-^ mice treated with IFM-514. In addition, NanoString gene expression analyses indicated that several fibrotic genes that were increased by MCD feeding were decreased by treatment with IFM-514, as shown in the heatmap (Figure 4F). Taken together, these data indicate that IFM-514 treatment markedly reduces key players in liver fibrosis.

### Hepatic Stellate Cell Activation and Portal Hypertension in MCD-Fed *ApoE* ^-/-^ Mice

**FIGURE 5:**
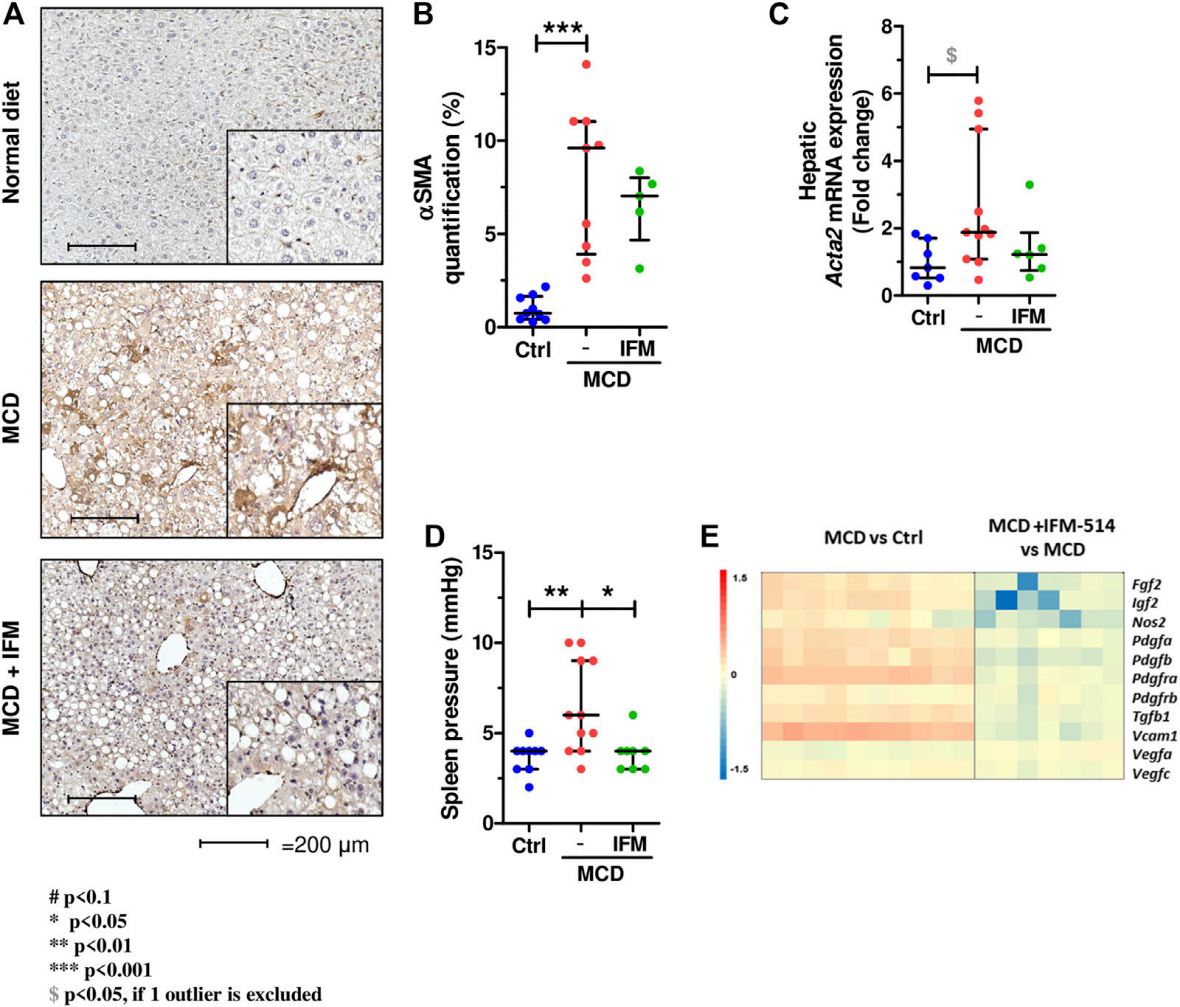
Hepatic stellate cell activation in MCD-fed *ApoE* ^*-/-*^ mice. *α*-smooth muscle actin immunostaining **(A)** with quantification **(B)** from IFM-514-treated MCD-fed *ApoE* ^*-/-*^ mice. *Acta2* mRNA expression **(C)**. Spleen pressure analyses **(D).** Heatmap for portal hypertension set of genes **(E).** All mRNA data were normalized to the expression of *18s*. Results are expressed as mean ± standard error of the mean (SEM); n = 10/group, #*p* < 0.1, *p < 0.05, **p < 0.01 and ***p < 0.001. ^$^ *p* < 0.05, if outliers are excluded. Scale bars = 200 μm. Abbreviations: CV, central vein; PV, portal vein; acta2, *α*-smooth muscle actin gene; *α*SMA, *α*-smooth muscle actin. See Supplementary Table 3 for Heatmap genes.

We next examined hepatic stellate cell activation (HSC) as an important mechanism for development of portal hypertension in addition to fibrosis (Abdulla et al., 2014), using quantitative immunostaining for *α*-smooth muscle actin (Acta2) (Figures 5A,B). Activation of HSC in MCD-fed mice was increased compared to controls, while IFM-514 treatment showed a tendency towards reduced *α*SMA expression. Similarly, the mRNA expression level of *Acta2* had a decreasing trend in IFM-treated MCD-fed *ApoE* ^-/-^ mice (Figure 5C). Portal pressure-measured in the spleen pulp-was significantly reduced in IFM-514-treated MCD-fed *ApoE* ^-/-^ mice (Figure 5D). Genes of key players in portal hypertension and activation of HSC were analyzed by NanoString gene expression (see heatmap, Figure 5E), showing a reduction of these genes with IFM-514 treatment.

### IFM-514 Reduces Hepatic Steatosis in MCD-Fed *ApoE* ^-/-^ Mice

**FIGURE 6:**
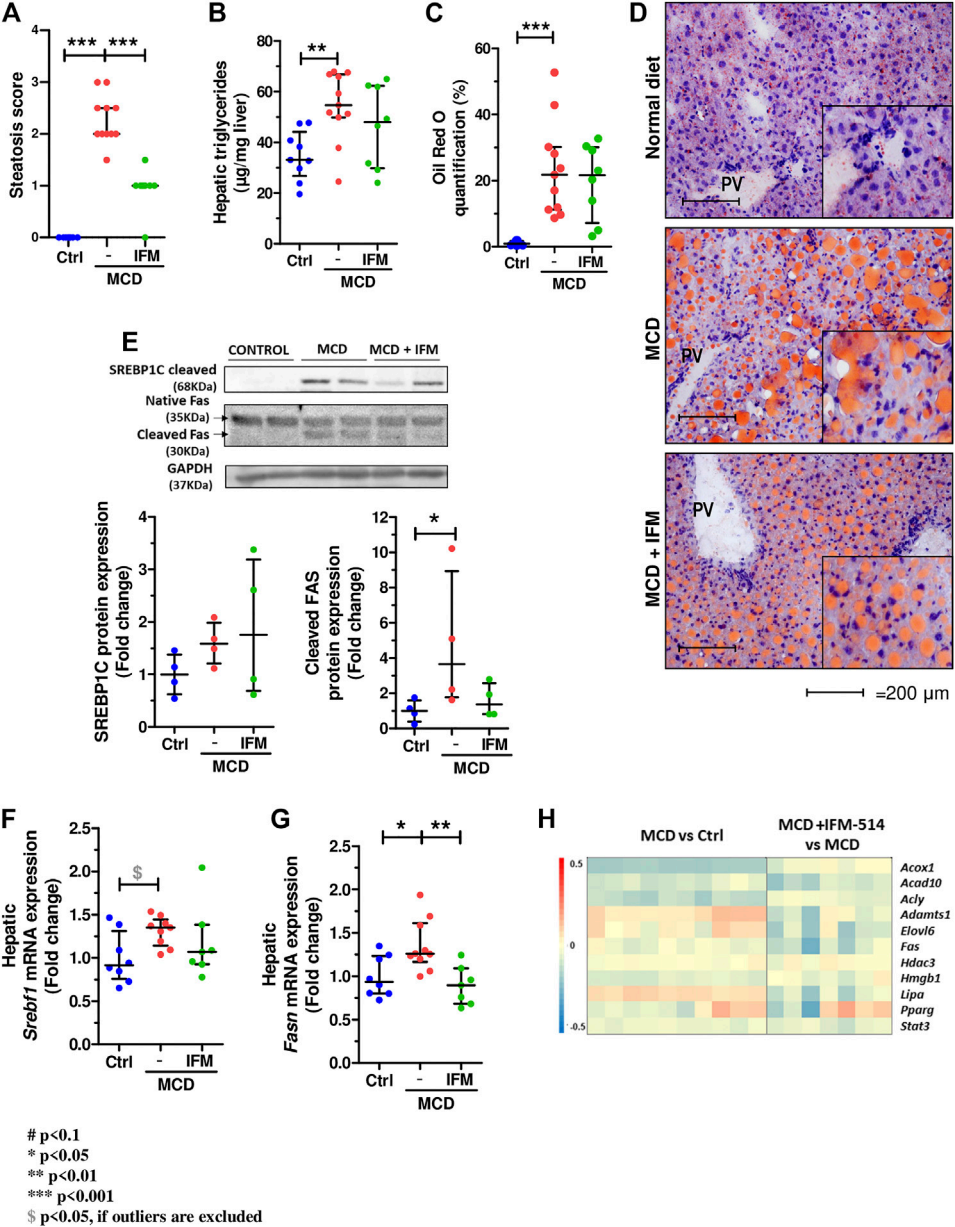
IFM-514 reduces hepatic steatosis in MCD-fed *ApoE* ^*-/-*^ mice. Steatosis score **(A)**. Hepatic triglycerides levels **(B)**. Oil red O staining and quantification as shown as percentage of the positive area **(C–D)**. Srebp1c, native Fas and cleaved Fas protein **(E)** and mRNA expression **(F–G)** from IFM-514-treated MCD-fed *ApoE* ^-/-^ mice. Heatmap for hepatic steatosis set of genes **(H)**. All mRNA data were normalized to the expression of *18s*. Results are expressed as the mean ± standard error of the mean (SEM); n = 10/group, *p < 0.05, **p < 0.01 and ***p < 0.001. Abbreviations: srebp1c; sterol regulatory element-binding protein 1c; *srebf1*, sterol regulatory element-binding transcription factor 1; *fas*, fatty acid synthase. See Supplementary Table 3 for Heatmap genes.

The steatosis score assessed by a liver pathologist demonstrated a significant reduction in mice treated with IFM-514 compared to vehicle treated mice (Figure 6A). Similarly, analysis of hepatic steatosis using quantitative hepatic triglycerides and oil red O staining revealed a trend towards a reduction in IFM-treated mice, although this was not significant, likely due to the persistence of numerous oil red O-positive small lipid droplets (Figures 6B–D). Similar trends were seen on protein and mRNA expression levels of central regulators of lipid homeostasis, sterol regulatory element binding factor 1 (Srebf1) and fatty acid synthase (Fasn), being significant for *Fasn* gene expression (Dinarello et al., 2012) (Figures 6E–G). The heatmap of the steatosis selected genes confirms the effect of IFM-514 treatment in MCD-fed mice (Figure 6H).

### IFM-514 Reduces Hepatic Gene Expression of Inflammasome-Related and Proinflammatory Cytokines in MCD-Fed *ApoE* ^-/-^ Mice

**FIGURE 7:**
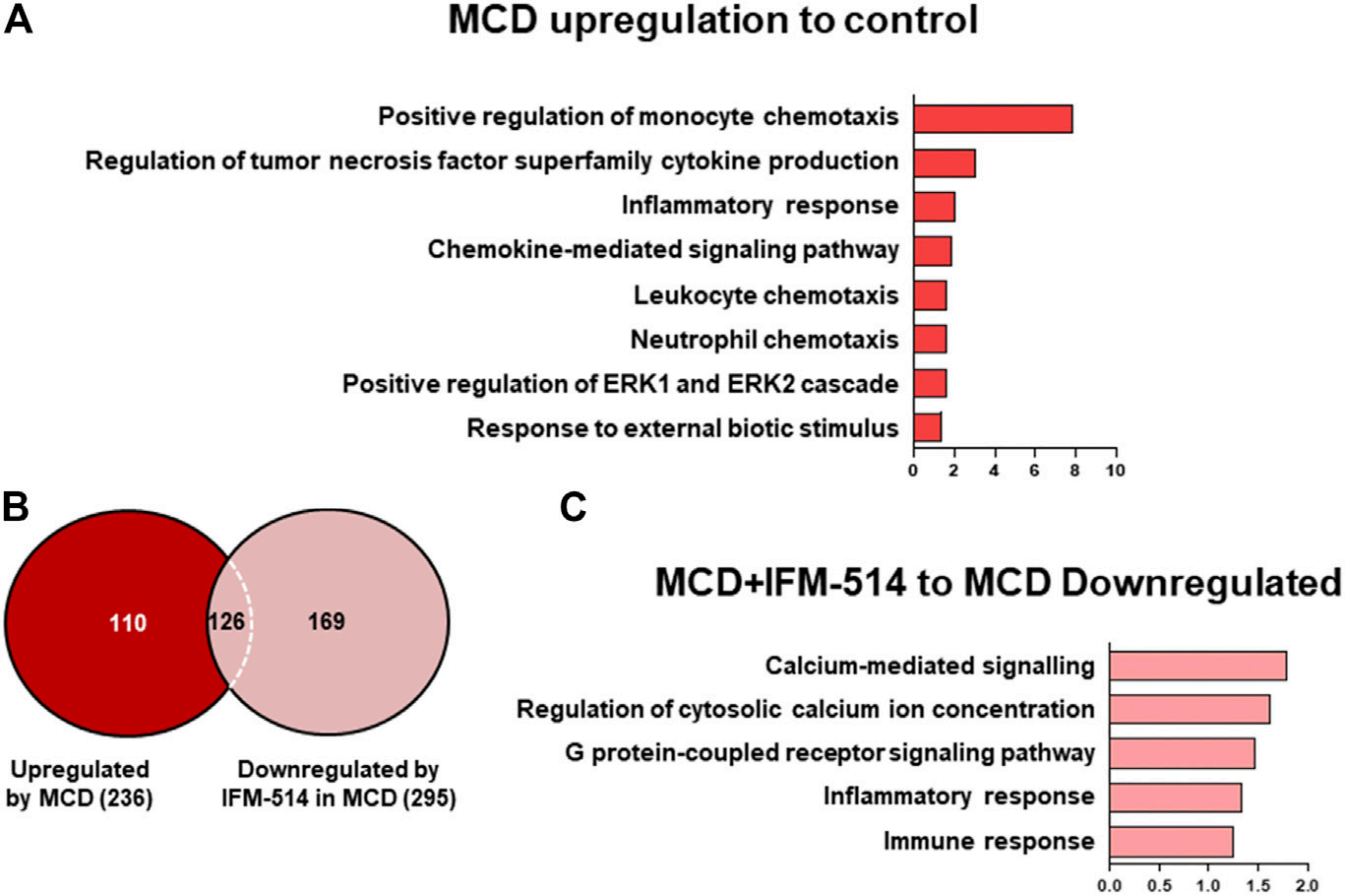
Transcriptomic analysis of IFM-514-treated MCD-fed *ApoE* ^*-/-*^ mice. Gene ontology (GO) analysis and fold enrichment of genes of biological process upregulated in MCD-fed **(A)**. Venn diagram of downregulated genes in IFM-514-treated MCD-fed **(B)**. Enriched GO terms for genes downregulated in IFM-514-treated MCD-fed **(C)**. The signaling pathways are based on statistical significance. Genes were considered significantly down expressed (*p* < 0.05) in IFM-treated mice by a minimum of 1.5 fold above that of vehicle-treated MCD-fed *ApoE* ^*-/-*^ mice.

To assess the extent of the genes differentially expressed following IFM-514 treatment in MCD-fed *ApoE* ^-/-^ mice, we analyzed the sets of genes upregulated in MCD-fed *ApoE* ^-/-^ mice (Figures 7A–C). To further characterize the upregulated pathways, we performed gene ontology (GO) analysis of all significantly upregulated genes in MCD-fed *ApoE* ^*-/-*^ mice. In biological processes, we found a one- to eight-fold enrichment in inflammation-related GO terms, such as regulation of tumor necrosis factor superfamily cytokine production and inflammatory response (Figure 7A). Through Venn analyses, a total of 236 genes were significantly upregulated in MCD-fed *ApoE* ^-/-^ mice (Figure 7B). Venn analyses revealed that a total of 126 genes specifically upregulated by MCD diet were found to be downregulated by IFM-514 (Figure 7B; Table 2). To examine the transcriptomic inflammatory signature in response to the IFM-514 treatment, we compared the sets of genes which were downregulated following IFM-514 treatment in MCD-fed *ApoE* ^-/-^ mice. GO analysis revealed enrichment for several genes related to calcium-mediated signaling, G protein-coupled receptor signaling pathway, inflammatory and immune responses in IFM-514-treated MCD-fed *ApoE* ^-/-^ mice (Figure 7C).

**TABLE 2:** IFM-514 changes the hepatic gene expression of inflammasome-related and proinflammatory cytokines in MCD-fed *ApoE* ^*-/-*^ mice. Table shows all genes significantly downregulated in IFM-514-treated MCD-fed mice.

Hepatic-specific downregulated gene set in IFM-514-treated in MCD mice								
Gene	*p*-value	x-Fold	Gene	*p*-value	x-Fold	Gene	*p*-value	x-Fold
2810417H13Rik	0.00667	−1.69064	Cxcr3	0.00068	−2.14185	Lpl	0.01570	−1.59407
Adam8	0.00011	−2.62183	Cxcr4	0.00319	−1.56080	Ltb	0.00006	−2.02311
Amica1	0.00011	−1.89605	Cybb	0.00019	−1.73405	Ltb4r2	0.02524	−1.71289
Anxa1	0.00870	−1.60182	Cytip	0.00017	−1.72377	Mmp12	0.00015	−4.50916
Areg	0.01426	−1.61760	Dusp2	0.00008	−2.06807	Mmp13	0.00068	−2.16102
Atf3	0.00372	−1.82585	Ear3	0.00000	−1.89868	Mpeg1	0.00011	−1.64865
Btk	0.00004	−1.58950	Fcgr1	0.00016	−1.61185	Ncf2	0.00003	−1.75356
C3ar1	0.00040	−1.65176	Fcgr3	0.00005	−1.75651	Nfatc2	0.00925	−1.57946
C5ar1	0.00300	−1.55740	Fcgr4	0.00165	−1.55248	Nlrp3	0.00041	−1.51407
Casp1	0.00018	−1.68863	Fgfr1	0.00169	−1.52777	Olr1	0.00057	−1.78952
Ccl17	0.00398	−1.67018	Flt3	0.00014	−2.02110	Pdgfb	0.00000	−1.83201
Ccl19	0.00039	−1.65110	Fpr2	0.01386	−1.54496	Plau	0.00058	−1.95671
Ccl2	0.00421	−2.19227	Fut4	0.00114	−2.06766	Psmb9	0.00493	−1.58966
Ccl22	0.00000	−2.95526	Gem	0.00001	−1.70950	Ptafr	0.00035	−1.84001
Ccl3	0.00237	−2.03073	Gpr65	0.00000	−1.95593	Ptprc	0.00083	−1.53368
Ccl4	0.00127	−2.61716	H2-Aa	0.00000	−2.89129	Retnla	0.00136	−6.02874
Ccnb2	0.00001	−2.47353	H2-Ab1	0.00000	−2.68355	Rgs1	0.02413	−1.69550
Ccr2	0.01111	−1.73233	H2-DMa	0.00001	−2.11027	Selplg	0.00063	−1.54297
Ccr7	0.00013	−1.99150	H2-DMb1	0.00014	−2.01112	Siglecf	0.00001	−2.47059
Ccr9	0.00000	−2.87416	H2-Eb1	0.00000	−2.66633	Sirpa	0.00024	−1.59139
Cd180	0.00101	−1.66737	Havcr2	0.00006	−1.90452	Syk	0.00000	−1.97188
Cd247	0.00020	−1.99446	Hdc	0.00035	−1.50346	Tgfb1	0.00001	−1.62258
Cd274	0.00005	−1.85501	Icam1	0.00005	−1.50435	Tlr13	0.00012	−1.85556
Cd40	0.00587	−1.53065	Icosl	0.00075	−1.58259	Tlr2	0.00000	−1.80107
Cd68	0.00003	−1.71272	Id3	0.00890	−1.54175	Tlr4	0.00071	−1.55125
Cd69	0.00014	−1.88949	Ikzf1	0.00020	−1.61504	Tlr6	0.00250	−1.50599
Cd74	0.00000	−2.45256	Il15	0.00373	−1.54588	Tlr7	0.00027	−1.85520
Cd80	0.00001	−1.96156	Il1b	0.02191	−1.64653	Tlr8	0.00000	−1.95168
Cd83	0.00899	−2.14652	Il1r2	0.00569	−2.68834	Tlr9	0.00017	−1.81612
Cd84	0.00009	−1.64947	Il1rn	0.00014	−1.90339	Tnf	0.00003	−2.76967
Cdc20	0.00000	−2.32831	Irf5	0.00017	−1.58824	Tnfaip3	0.00665	−1.77645
Cdh4	0.00324	−1.57430	Irf8	0.00003	−1.83892	Tnfaip8	0.00012	−1.53840
Clec5a	0.00002	−2.26914	Isg15	0.01062	−1.68263	Tnfrsf11a	0.00000	−1.76821
Clec7a	0.00003	−2.82955	Itga4	0.00001	−1.98049	Top2a	0.01684	−1.70561
Clec9a	0.01630	−1.56250	Itgal	0.00002	−1.79662	Trem2	0.00008	−2.01950
Ctsd	0.00001	−1.57586	Itgax	0.00000	−3.08656	Tspan8	0.00348	−1.84056
Ctss	0.00000	−2.23524	Itgb2	0.00002	−1.79929	Tyrobp	0.00021	−1.61424
Cx3cr1	0.00011	−2.38809	Kif20a	0.00001	−2.58010	Usp18	0.00012	−1.83401
Cxcl10	0.00001	−2.98911	Laptm5	0.00000	−1.82459	Vav1	0.00029	−1.52158
Cxcl16	0.00023	−1.58510	Lat2	0.00004	−2.22728	Vcam1	0.00017	−1.83038
Cxcl2	0.00007	−2.36479	Lgals3	0.00001	−2.09902	Vsir	0.00021	−1.62241
Cxcl9	0.00843	−2.14758	Lipa	0.00002	−1.66341	Was	0.00009	−1.76558

Thus, IFM-514 clearly reduced the expression of several genes specifically upregulated in the MCD model, not limited to inflammasome function, but related to inflammation in general. All in all, these results strongly support that the primary targets of the NLRP3 blockade are liver inflammation and fibrosis, with a mild effect on hepatic steatosis in MCD-fed *ApoE* ^-/-^ mice.

### IFM-514 Effect on Hepatic Inflammation and Fibrosis in WD-Fed *ApoE* ^-/-^ Mice

Since MCD-fed mice recapitulate the histology of human NAFLD (hepatic steatosis, and fibrosis) but lack the metabolic component (obesity, high cholesterol and diabetes) to assess the impact of metabolic imbalances in NLRP3 activation in NASH, *ApoE* ^-/-^ mice were fed with WD. 12-week-old *ApoE* ^-/-^ mice were fed for a total of 7 weeks with WD diet. After 3 weeks of diet-induced injury, mice received i. p. injections of IFM-514 (100 mg/kg) or vehicle for 5 days/week for a further 4 weeks (Supplementary Figure 2A). IFM-514 was measured by HPLC-MS in liver and serum 8 h after the last dose of IFM-514 in WD-fed *ApoE* ^-/-^ mice which was rather low compared to MCD fed *ApoE* ^-/-^ (Supplementary Figures 2B–C). IFM-514-treated NASH *ApoE* ^-/-^ mice had similar circulating IL-1*β* and IL-1*α* as vehicle-treated NASH *ApoE* ^-/-^ mice (Supplementary Figures 2D–E).

We further investigated the effect of IFM-514 treatment on inflammation and fibrosis in WD-fed *ApoE* ^-/-^ mice. No difference in inflammation score between *ApoE* ^-/-^ and WD fed *ApoE* ^-/-^ mice was found (Supplementary Figure 3B), but WD induced an increase in NAS score (Supplementary Figure 3C). In addition, WD significantly increased *Tnf-α* gene expression (Supplementary Figure 3E) while IFM-514 showed a tendency towards reduction in some inflammatory genes, being significant for Ccl1 and Ccl28 (Supplementary Figures 3F–G). Liver fibrosis was assessed by sirius red staining and hepatic hydroxyproline content, which also tend to decrease by the IFM-514 treatment (Supplementary Figures 3A,C,E). Activation of hepatic stellate cells assessed by *α*-sma staining (Supplementary Figures 4B,D), was significantly increased in WD fed *ApoE* ^-/-^ mice, and showed a tendency to decrease after the IFM-514. Similar effects showed spleen pressure (Supplementary Figure 4F) and the heatmaps of fibrotic and portal hypertension related genes (Supplementary Figure 4G–H).

## Discussion

Currently, therapies targeting NLRP3-dependent cytokines, i.e. canakinumab, an IL-1*β*-neutralizing antibody, and rilonacept, a soluble receptor that binds IL-1*β* and IL-1*α*, are applied in humans. However, they have notable immunosuppressive disadvantages compared to selective NLRP3 antagonism (Dinarello et al., 2012; Ridker et al., 2017). This is of particular relevance when considering that in a targeted anti-NLRP3 therapy, other pathogen-recognizing inflammasomes can be engaged to produce IL-1*β*, thus reducing the risk of immune suppression and opportunistic infections. Supporting this idea, similar concentrations of circulating IL-1*β* and IL-1*α* detected in IFM-514-treated vs untreated NASH mice might lower the risk of immunosuppressive effects of this anti-inflammasome therapy, rendering the IFM-514 treatment very suitable in NASH.

A recent study investigated the effect of the NLRP3 antagonist CP-456,773 (Jiang et al., 2017) (also called MCC950 or CRID3) on the development of NASH in dietary models (Mridha et al., 2017). Similar to our data, CRID3 caused a significant reduction in hepatic infiltration of macrophages and neutrophils and modulated fibrotic progression of steatohepatitis in MCD-fed as well as foz/foz mice fed with atherogenic diet. These effects correlated with a significant reduction of NLRP3 activation in the liver, suggesting an important role for NLRP3 in the progression of NASH. Nevertheless, further work is still required regarding the precise molecular target and safety of CRID3. Indeed, liver toxicity has been observed in humans, probably due to either the high dose of CRID3 (1,200 mg per day) or a reactive metabolite of its furan moiety or both (Mangan et al., 2018). The latter two are well-known causes of drug-induced liver toxicity. By contrast, in our study, the daily administration of 100 mg/kg of IFM-514 reached adequate concentrations in liver and serum. These concentrations are low enough not to induce liver toxicity, but still sufficient to inhibit NLRP3 *in vitro*, as shown by the observed effects of IFM-514 in the *in vivo* LPS-PD/PK studies.

NAFLD is generally considered the hepatic manifestation of the metabolic syndrome and its predominant underlying risk factors appear to be increased body weight and obesity. However, NAFLD can occur in lean subjects with a body mass index <25 kg/m^2^ (Voss et al., 2011), suggesting that other predisposing factors and inherited disorders play a critical role in lean NAFLD patients, which is an important consideration in Asian populations (Wong et al., 2018). In the MCD diet, the absence of methionine leads to increased hepatic inflammation, fibrosis, liver damage and macrovesicular steatosis (Caballero et al., 2010). Therefore, MCD-fed mice show severe steatohepatitis but is not caused by metabolic syndrome or insulin resistance and show weight loss and increased mortality (Larter and Yeh, 2008). For that reason, this study was conducted in *ApoE* ^-/-^ mice, which have a genetic predisposition to develop chronic inflammation (Harja et al., 2008), including metabolic syndrome (Schierwagen et al., 2015) as a useful tool to explore the therapeutic effects of a novel selective NLRP3 antagonist in lean NAFLD.

There is compelling evidence that NLRP3 inflammasome acts as a mediator of inflammation, lipotoxicity, and fibrosis (Duewell et al., 2010; Bracey et al., 2014; Mehta et al., 2014). Also, NLRP3 has been identified as a central insult sensor that triggers and sustains disease driven by metabolic dysfunction and fibrosis following either acute tissue injury or chronic inflammation (Christ et al., 2018). Similarly, NLRP3 activation occurs when oxidation of LDL, cholesterol, and fats are increased (Shi et al., 2006; Saberi et al., 2009). Thus, it is likely that blockage of the NLRP3 signaling pathway will decelerate the progression from NAFLD to NASH. Therefore, we tested a novel selective NLRP3 antagonist in a dietary *in vivo* study to reduce fibrosis and inflammation in NASH-induced *ApoE* ^-/-^ mice.

The essential findings of the present study are that an anti-NLRP3 approach in NASH can arrest established liver fibrosis and chronic inflammation in *ApoE* ^-/-^ mice. As for liver fibrosis, this was confirmed by robust readouts. Importantly, the ability of IFM-514 to reduce liver fibrosis is highly relevant as liver fibrosis is associated with adverse liver outcomes in NASH (Angulo et al., 2015). Concerning hepatic chronic inflammation, the NanoString panel and the analysis of the specific gene set enrichment accompanied by reduced Tnf*α* mRNA expression clearly demonstrate a reduction in cytokine- and inflammasome-related genes by IFM-514. These results indicate that IFM-514 exerts both anti-fibrotic and anti-inflammatory effects in NASH *ApoE* ^-/-^ mice.

Several lines of evidence suggest that a specific NLRP3 antagonist could be an effective novel therapy in NASH for several reasons. First, the transition from NAFLD to NASH correlates with the accumulation of hepatic cholesterol crystals, a known NLRP3 trigger (Duewell et al., 2010; Ioannou et al., 2013). Second, genetic NLRP3 deficiency (Stienstra et al., 2011; Vandanmagsar et al., 2011; Wree et al., 2014a) as well as a specific NLRP3 antagonist (Mridha et al., 2017) halt the progression of NAFLD into NASH. Third, the gain-of-function NLRP3 knock-in mice exhibited enhanced NASH-induced fibrosis when fed with MCD (Wree et al., 2014a). Collectively, these data suggest that NLRP3 inhibitors are potential targets in obesity-induced inflammation and insulin resistance (Vandanmagsar et al., 2011), NAFLD (Henao-Mejia et al., 2012; Wree et al., 2014b) and NASH (Mridha et al., 2017). IFM-514 inhibited NLRP3 inflammasome and the subsequent caspase-1 proteolytic activation, thus preventing the development of NASH in *ApoE* ^-/-^ mice.

Our study has several limitations. The treatment regimen, although based on previous data using NLRP3 inhibitor (Mridha et al., 2017), may have been more efficient if the treatment would be administrated daily. In addition, the lower exposure from IP dosing in those mice may be the reason for reduced efficacy. Moreover, MCD diet in *ApoE* ^-/-^ mice is not the standard model of NASH, but still a model with high liver inflammation and fibrosis, and without substantial weight loss (Schierwagen et al., 2015). Finally, although NLRP3 inhibition with IFM-514 reduces lipogenic genes, it fails to decrease oil red staining in the livers of treated animals. One explanation may be that the NLRP3 inhibition decreases the inflammatory response and thereby the lipogenic stress in a second step.

Our data indicate that the beneficial effects of IFM-514 against steatohepatitis in the MCD *ApoE* ^-/-^ model were due to reduction of hepatic inflammation and fibrosis with marginal changes in lipid accumulation. Thus, these findings demonstrate a link between NLRP3 inflammasome activation and the progression to NASH.

## Conclusion

IFM-514 reduced liver inflammation and fibrosis, with mild effect in liver steatosis in MCD-fed *ApoE* ^-/-^ mice. These data suggest that blocking NLRP3 might be an attractive therapeutic approach for lean NASH patients.

## Methods

### Formulation of IFM-514

NLRP3 inhibitor, IFM-514, was kindly provided by the manufacturer (IFM Therapeutics, Boston, MA, United States) and it was supplied in the form of a sodium salt that is soluble in water at concentrations up to 2 mM. To prepare a suspension for i. p. or oral (100 mg/kg, 10 ml/kg) dosing, the appropriate amount of dry powder was weighed out and added to an appropriate volume of 0.5% carboxymethylcellulose (CMC) in PBS to obtain a 10 mg/ml suspension. The physicochemical properties of IFM-514 are shown in Supplementary Table 1.

### *In Vitro* Experiments

To analyze the *in vitro* potency and selectivity of IFM-514, mouse and human inflammatory cells were used. To determine the activity of IFM-514 on gramicidin-induced release of IL-1*β* in cell lines, PMA-differentiated human THP-1 cells were treated with IFM-514 for 1 h and then stimulated with gramicidin (5 μM) for 2 h; an immortalized murine macrophage cell line was primed with LPS (200 ng/ml) for 2 h, treated with IFM-514 for 1 h and then stimulated with gramicidin (2 μM) for 2 h. Primary human CD14 ^+^ monocytes were treated with GM-CSF and IL-4 for 6 days to induce differentiation to macrophages. The cells were pre-treated with IFM-514 for 1 h, primed with LPS for 2 h and then triggered with gramicidin for 2 h. In addition, BALB/c bone marrow-derived cells were treated with M-CSF for 6 days to induce differentiation to macrophages. After an overnight rest, cells were pre-treated with compounds for 1 h, primed with LPS (10 ng/ml) for 3 h and stimulated with gramicidin (5 μM final) for 1 h. In all cases, the activity of NLRP3 was evaluated by measuring the production of IL-1*β* in the supernatant. In all experiments, the IL-1*β* production was analyzed by homogeneous time resolved fluorescence (HTRF) in cell-free supernatant.

### Mice

Female C57BL/6 mice used for the acute lipopolysaccaride (LPS)-induced peritoneal inflammation model (*p*K/PD) were obtained from Jackson Laboratory. Mice were kept under specific pathogen-free conditions and provided with food and water ad libitum. The animal studies were conducted under protocols approved by the TSRL Institutional Animal Care and Use Committee (IACUC) (application number EFF-21001-02).

A total number of 30 male Apolipoprotein E-deficient mice (*ApoE* ^-/-^ mice) were used for all studies. *ApoE* ^-/-^ mice are predisposed to hypercholesterolemia, atherosclerosis, and obesity. All experiments were performed in accordance with the German animal protection law and statutory guidelines of the animal care facility (Haus für experimentelle Therapie, University Clinics Bonn, Germany), and approved by the North Rhine-Westphalia State Agency for Nature, Environment, and Consumer Protection (LANUV, file reference LANUV NRW, 84-02.04.2014.A137).

### LPS-Induced Cytokine Release in C57BL/6 Mice

C57BL/6 mice (8–10 weeks of age, Jackson Laboratories) in groups of 10 were pre-dosed orally 1 h before study start with IFM-514 or vehicle (0.5% CMC in water) at various concentrations in a total volume of 10 ml/kg. The mice were then returned to their cage and allowed food and water ad libitum. After 1 h, mice were then injected IP with 20 mg/kg of LPS (0.1 ml in saline) and again returned to their cages. After an five additional hours (t = 6 h post drug), blood was collected for cytokine analysis by ELISA and quantification of IFM-514 was performed by HPLC using standard reverse-phase conditions.

### Induction of NASH With Liver Fibrosis in Mice

10–12-week-old *ApoE* ^-/-^ male and female C57BL/6 mice were fed ad lib for 7 weeks with a methionine/choline deficient (MCD) diet (Diet#E15653-94, Ssniff Spezialdiäten GmbH, Soest, Germany) or with a high-fat cholesterol-rich Western diet (WD) (Diet#S0279-S011, 1.25% cholesterol, Ssniff Spezialdiäten GmbH, Soest, Germany). In MCD-fed mice, hepatic steatosis became histologically evident after 10 days and fibrosis after 8–10 weeks (Schierwagen et al., 2015, 2016). The MCD model shows severe steatohepatitis but is not caused by overnutrition or associated with insulin resistance (Larter and Yeh, 2008). After 3 weeks of diet-induced injury, mice were injected i. p. with 100 mg/kg/day of an NLRP3 antagonist IFM-514 (IFM Therapeutics Inc., Boston, United States) (100 mg/kg body weight) or vehicle (0.5% carmellose) every day, 5 days/week for a further 4 weeks. Serum and liver were collected for histological and molecular readouts of fibrosis, inflammation and steatosis. Portal pressure measured invasively in the spleen pulp, a cannula made from a 25-gauge needle connected to a saline-filled manometer was inserted into the spleen pulp, as previously described (Ackerman et al., 2008). The catheters were connected to a pressure transducer (Hugo Sachs Elektronik, March-Hugstetten, Germany) for blood pressure measurement. Splenic pulp pressure was measured as an index of portal venous pressure (Ppv). For assessment of liver injury, inflammation, fibrosis and steatosis, liver samples were fixed in 10% neutral-buffered formalin and paraffin embedded. Liver sections were stained with H&E and Sirius red (SR). Fibrosis severity was determined by SR morphometry and hepatic 4-hydroxyproline content as previously described (Schierwagen et al., 2015, 2016). To demonstrate steatosis, hepatic triglycerides were measured using an enzyme-linked colorimetric assay TG liquicolor mono (Human Diagnostics, Wiesbaden, Germany) and fresh-frozen hepatic sections were stained with oil red O for neutral fat.

### Immunohistochemical Staining

Immunohistochemical staining for *α*SMA was performed in paraffin-embedded sections (5 μm). The sections were incubated with a mouse-anti-SMA antibody (Actin clone 1A4; Dako, Hamburg, Germany). Thereafter, biotinylated goat-anti-mouse (Dako, Hamburg, Germany) secondary antibody was used. Finally, sections were counterstained with hematoxylin.

### Pathology

Blind analysis by a liver pathologist was carried out to determine the activity scores according to Kleiner et al. (Kleiner et al., 2005). The NAFLD Activity Score (NAS) was calculated as the sum of scores for steatosis, lobular inflammation, pigmented macrophages, and fibrosis.

### Mouse Serum Chemokines

Blood was collected at the time of euthanasia, maintained at RT for 30 min to retract the clot and centrifuged at 2,000 rpm for 5 min for serum collection. Cytokines in mouse sera were measured with the BioPlex mouse cytokine assay on a Bio-Plex 200 system powered by Luminex xMAP Technology. The analysis was performed according to the manufacturer’s protocol.

### Gene Expression Analysis by NanoString Technology

We determined hepatic expression of specific mRNAs by reverse-transcriptase quantitative PCR (RT-qPCR), following extraction of total RNA from liver tissue. Primer sequences are shown in Supplementary Table 2. Expression of mRNA was relative to 18 s.

The prevalence of hepatic innate inflammation was evaluated by determining expression of a panel of mouse myeloid innate immunity (770 immune-related genes) using the NanoString technology (NanoString Technologies, Seattle, United States) according to manufacturer’s instructions. Briefly, 200 ng of total RNA was hybridized, quantified, and loaded into Partek Genomics Suite (Partek Inc. St. Louis, MO, United States). The gene enrichment analysis was performed using Gene Ontology and Consensus Pathway DB. Heatmaps were generated using the gplots library in R. Genes were considered significantly down expressed (*p* < 0.05) in IFM-treated mice by a minimum of 1.5 fold above that of vehicle-treated MCD-fed *ApoE* ^-/-^ mice, using a hypergeometric test with FDR <0.05. Venn diagram was used to intersect the predicted target genes and the distribution of the 1.5-fold differentially expressed genes, showing the overlap in genes significantly upregulated and downregulated.

### Western Blotting

Protein levels were analyzed by Western blot as described previously (Granzow et al., 2014; Brol et al., 2019). Briefly, snap-frozen livers were homogenized and diluted. Protein quantification was performed using a colorimetric BCA protein assay kit (Cat 23225, Thermo Fisher Scientific Inc., IL, United States). Forty micrograms of protein samples was subjected to SDS-PAGE under reducing conditions (10% gels), and proteins were blotted on nitrocellulose membranes. The membranes were blocked and incubated with primary antibody against collagen 1*α*1 (SC-12895; Santa Cruz Biotechnology, Santa Cruz, CA), srebp1c (ab28481, Abcam), fas (ab82419, Abcam), caspase-1 (sc-56036, Santa Cruz Biotechnology, Santa Cruz, CA), IL-1*β* (NB600-633). Glyceraldehyde-3-phosphate dehydrogenase (GAPDH) served as an endogenous control (sc-47724; Santa Cruz Biotechnology, Santa Cruz, CA). Membranes were incubated with the corresponding secondary antibody, and blots were developed using enhanced chemiluminescence. Protein quantification was performed by ImageJ (version 1.51q, NIH, United States) and results were corrected for GAPDH levels.

### Statistical Analysis

Statistical analyses among groups were performed using Prism V.5.0 (GraphPad, San Diego, CA). Comparisons between two groups were done by non-parametric Mann-Whitney U t-tests and one-way ANOVA followed by Tukey’s Multiple Comparison test were used for statistical comparisons between the three groups, with *p* < 0.05 considered as significant (#< 0.1. *p < 0.05, **p < 0.01, ***p < 0.001, ****p < 0.0001). $ *p* < 0.05, if the point is excluded as an outlier. Data were expressed as mean ± SEM. All experiments were performed in triplicate at least four times and a representative image or blots are shown on the manuscript. For transcriptome analysis, statistical parameters were computed between groups, and results are shown as log2-fold change and visualized by heatmaps. *p*-values were calculated using paired *t*-test and corrected according to the adaptive Benjamini-Hochberg procedure. A FDR-adjusted *p*-value below 0.05 was considered statistically significant.

## Data Availability

The original contributions presented in the study are included in the article/Supplementary Material, further inquiries can be directed to the corresponding author.
