# Supplementary figures and images for: The Specific NLRP3 Antagonist IFM-514 Decreases Fibrosis and Inflammation in Experimental Murine Non-Alcoholic Steatohepatitis
Source: Front Mol Biosci. 2021 Aug 13;8:715765. doi: 10.3389/fmolb.2021.715765 (PMC8425476; doi:10.3389/fmolb.2021.715765)

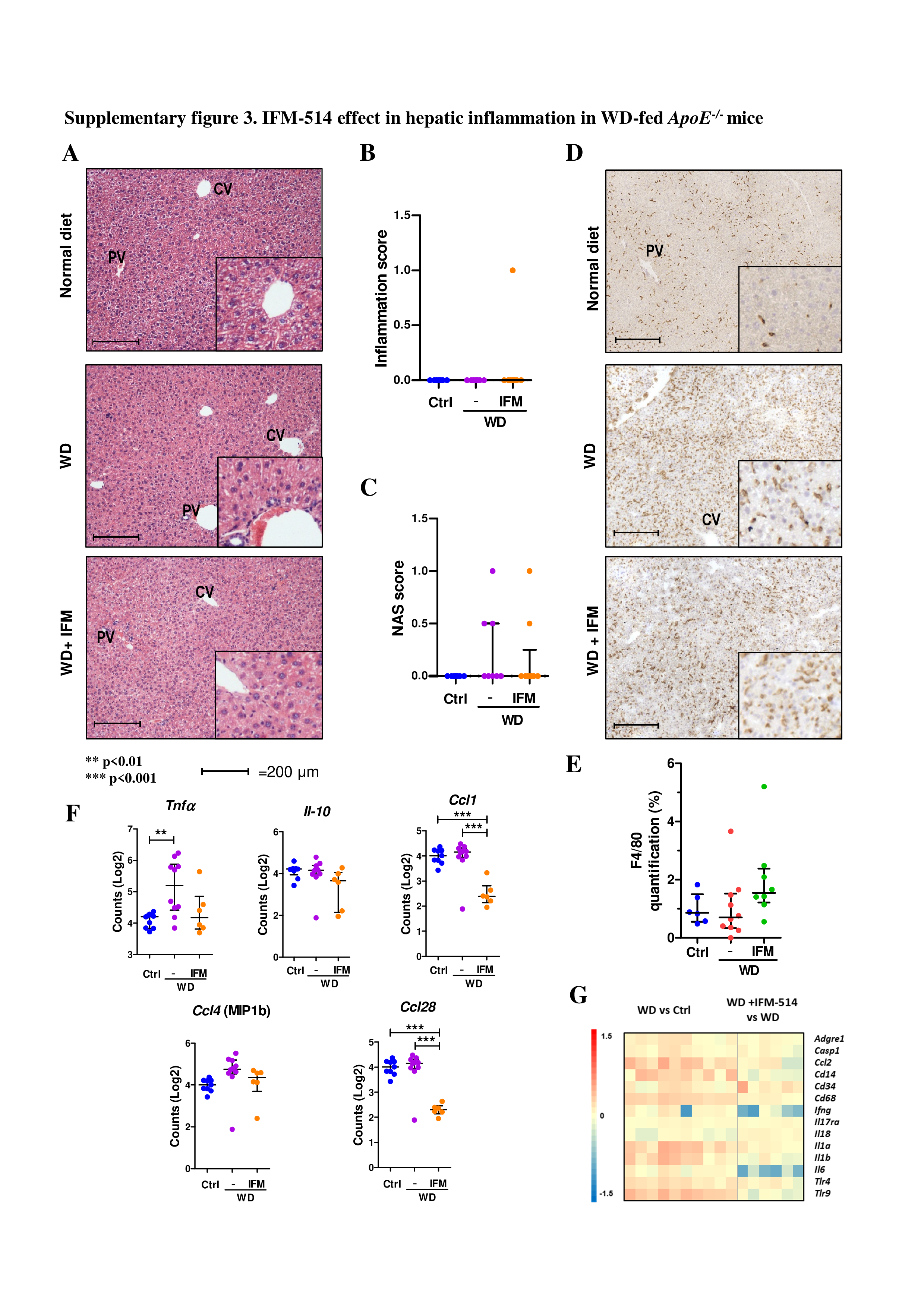

Supplement: Supplementary file 1 [file Image3.TIF]

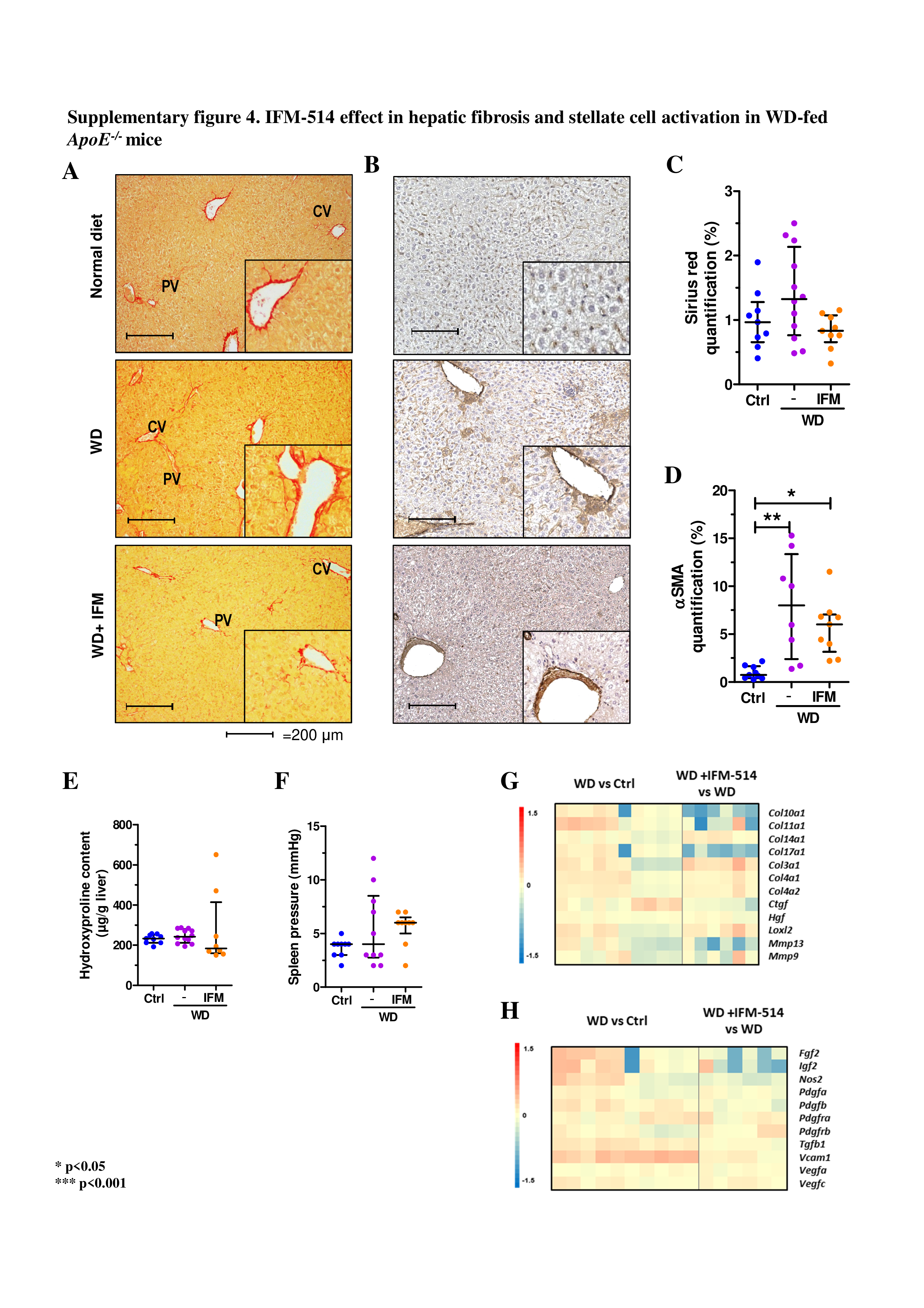

Supplement: Supplementary file 2 [file Image4.TIF]

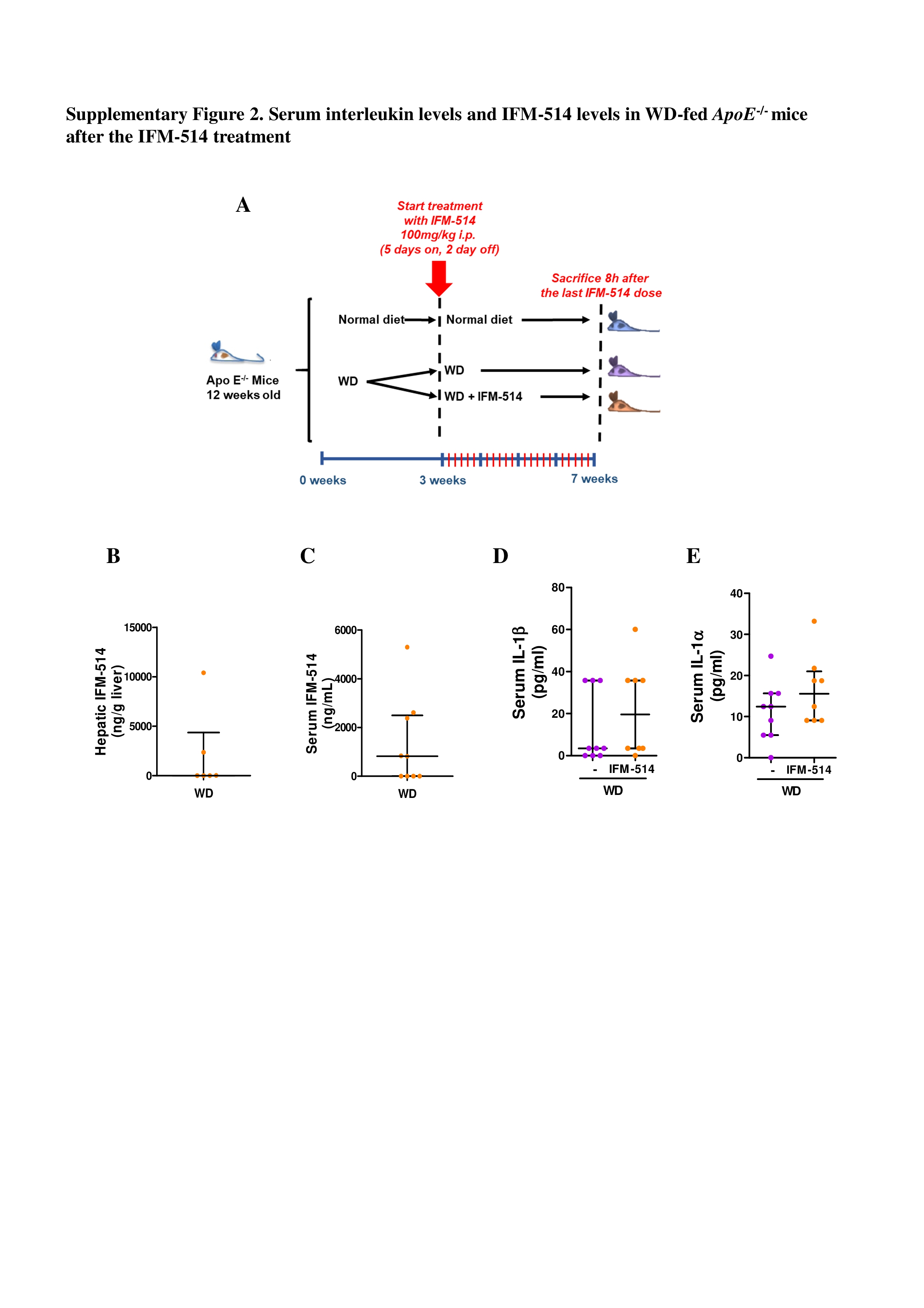

Supplement: Supplementary file 3 [file Image2.JPEG]

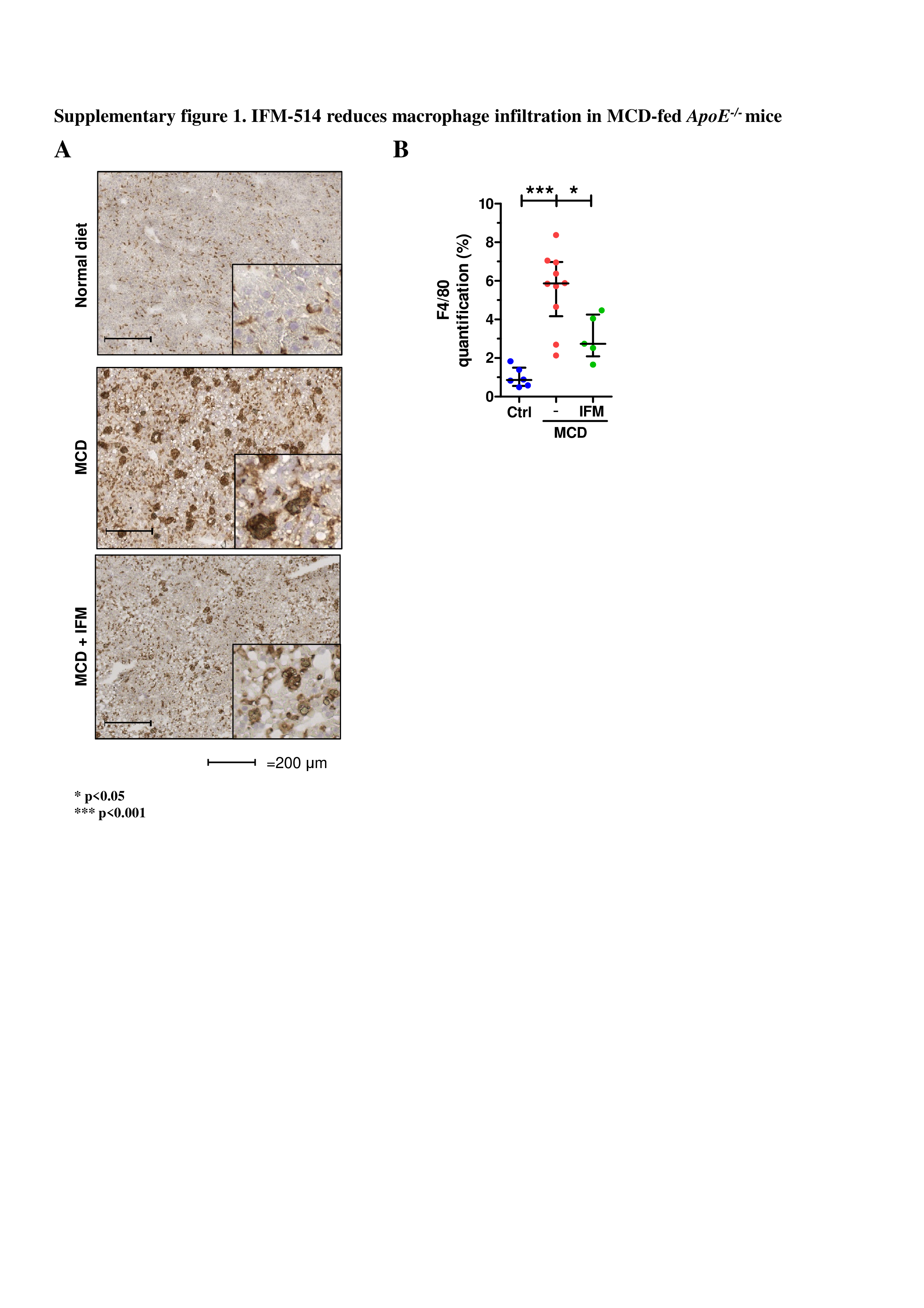

Supplement: Supplementary file 4 [file Image1.TIF]
