# Supplementary material for: The Specific NLRP3 Antagonist IFM-514 Decreases Fibrosis and Inflammation in Experimental Murine Non-Alcoholic Steatohepatitis
Source: Front Mol Biosci. 2021 Aug 13;8:715765. doi: 10.3389/fmolb.2021.715765 (PMC8425476; doi:10.3389/fmolb.2021.715765)
Supplement: Supplementary file 5 [file DataSheet1.docx]

Supplementary Material

1. **Supplementary Tables**

**Table S1.** Single dose pharmacokinetics studies of IFM-514 in C57BL/6 mice:

| **Route** | **Dose (mg/Kg)** | **C_max_ (μM)** | **AUC_inf_ (h*μM)** | **T_1/2_ (h)** |
| --- | --- | --- | --- | --- |
| IP | 100 | 480 | 2975 | 4.6 |
| PO | 100 | 1274 | 3829 | 2.6 |

**Table S2.** List of validated TaqMan^®^ gene expression assays

| **Gene** | **Assay ID** |
| --- | --- |
| *acta2* | Mm00725412_s1 |
| *col1a1* | Mm00801666_g1 |
| *srebf1* | Mm00550338_m1 |
| *fasn* | Mm01204974_m1 |

**Table S3.** List of gene abbreviations

***2810417H13Rik,*** RIKEN cDNA 2810417H13 gene

***Acad10,*** acyl-CoA dehydrogenase family member 10

***Acly,*** ATP citrate lyase

***Acox1,*** acyl-CoA oxidase 1

***Adam8,*** a Disintegrin and metalloproteinase domain-containing protein 8

***Adamts1,*** a disintegrin and metalloproteinase with thrombospondin motifs 1

***Adgre1,*** adhesion G protein-coupled receptor E1

***Amica1,*** junctional adhesion molecule-like

***Anxa1,*** annexin A1

***Areg,*** amphiregulin

***Atf3,*** activating transcription factor 3

***Btk,*** Bruton's tyrosine kinase

***C3ar1,*** complement component 3a receptor 1

***C5ar1,*** complement component 5a receptor 1

***Casp1,*** caspase 1

***Ccl17,*** chemokine (C-C motif) ligand 17

***Ccl19,*** chemokine (C-C motif) ligand 19

***Ccl2,*** chemokine (C-C motif) ligand 2

***Ccl22,*** chemokine (C-C motif) ligand 22

***Ccl3,*** chemokine (C-C motif) ligand 3

***Ccl4,*** chemokine (C-C motif) ligand 4

***Ccnb2,*** G2/mitotic-specific cyclin-B2

***Ccr2,*** C-C chemokine receptor type 2

***Ccr7,*** C-C chemokine receptor type 7

***Ccr9,*** C-C chemokine receptor type 9

***Cd14,*** [cluster of differentiation](https://en.wikipedia.org/wiki/Cluster_of_differentiation) 14

***Cd180,*** [cluster of differentiation](https://en.wikipedia.org/wiki/Cluster_of_differentiation) 180

***Cd247,*** [cluster of differentiation](https://en.wikipedia.org/wiki/Cluster_of_differentiation) 247

***Cd274,*** [cluster of differentiation](https://en.wikipedia.org/wiki/Cluster_of_differentiation) 274

***Cd34,*** [cluster of differentiation](https://en.wikipedia.org/wiki/Cluster_of_differentiation) 34

***Cd40,*** [cluster of differentiation](https://en.wikipedia.org/wiki/Cluster_of_differentiation) 40

***Cd68,*** [cluster of differentiation](https://en.wikipedia.org/wiki/Cluster_of_differentiation) 68

***Cd69,*** [cluster of differentiation](https://en.wikipedia.org/wiki/Cluster_of_differentiation) 69

***Cd74,*** [cluster of differentiation](https://en.wikipedia.org/wiki/Cluster_of_differentiation) 74

***Cd80,*** [cluster of differentiation](https://en.wikipedia.org/wiki/Cluster_of_differentiation) 80

***Cd83,*** [cluster of differentiation](https://en.wikipedia.org/wiki/Cluster_of_differentiation) 83

***Cd84,*** [cluster of differentiation](https://en.wikipedia.org/wiki/Cluster_of_differentiation) 84

***Cdc20,*** [cluster of differentiation](https://en.wikipedia.org/wiki/Cluster_of_differentiation) 20

***Cdh4,*** cadherin-4

***Clec5a,*** C-type lectin domain family 5 member A

***Clec7a,*** C-type lectin domain family 7 member A

***Clec9a,*** C-type lectin domain family 9 member A

***Col10a1,*** collagen type 10 alpha-1

***Col11a1,*** collagen type 11 alpha-1

***Col14a1,*** collagen type 14 alpha-1

***Col17a1,*** collagen type 17 alpha-1

***Col3a1,*** collagen type 3 alpha-1

***Col4a1,*** collagen type 4 alpha-1

***Col4a2,*** collagen type 4 alpha-2

***Ctgf,*** connective tissue growth factor

***Ctsd,*** cathepsin D

***Ctss,*** cathepsin S

***Cx3cr1,*** CX3C chemokine receptor 1

***Cxcl10***, C-X-C motif chemokine ligand 10

***Cxcl16***, C-X-C motif chemokine ligand 16

***Cxcl2***, C-X-C motif chemokine ligand 2

***Cxcl9***, C-X-C motif chemokine ligand 9

***Cxcr3,*** chemokine receptor CXCR3

***Cxcr4,*** chemokine receptor CXCR4

***Cybb,*** [cytochrome b](https://en.wikipedia.org/wiki/Cytochrome_b-245,_alpha_polypeptide) beta

***Cytip,*** cytohesin-interacting protein

***Dusp2,*** dual specificity protein phosphatase 2

***Ear3,*** eosinophil cationic-type ribonuclease 3

***Elovl6,*** elongation of very long chain fatty acids protein 6

***Fas,*** fatty acid synthase

***Fcgr1,*** low affinity immunoglobulin gamma Fc region receptor 1

***Fcgr3,*** low affinity immunoglobulin gamma Fc region receptor 3

***Fcgr4,*** low affinity immunoglobulin gamma Fc region receptor 4

***Fgf2,*** fibroblast growth factor 2

***Fgfr1,*** fibroblast growth factor receptor 1

***Flt3,*** [fms related receptor tyrosine kinase 3](https://www.ncbi.nlm.nih.gov/gene/2322)

***Fpr2,*** [formyl peptide receptor 2](https://www.ncbi.nlm.nih.gov/gene/2358)

***Fut4,*** fucosyltransferase 4

***Gem,*** mitogen-induced gene

***Gpr65,*** G protein-coupled receptor 65

***H2-Aa,*** histocompatibility 2, class II antigen A, alpha

***H2-Ab1,*** histocompatibility 2, class II antigen A, beta 1

***H2-DMa,*** histocompatibility 2, class II, locus Dma

***H2-DMb1,*** histocompatibility 2, class II, locus DMb1

***H2-Eb1,*** histocompatibility 2, class II antigen E beta

***Havcr2,*** hepatitis A virus cellular receptor 2

***Hdac3,*** histone deacetylase 3

***Hdc,*** histidine decarboxylase

***Hgf,*** hepatocyte growth factor

***Hmgb1,*** high mobility group box 1

***Icam1*,** intercellular adhesion molecule 1

***Icosl,*** inducible T cell costimulator ligand

***Id3,*** inhibitor of dna binding 3

***Ifng,*** interferon gamma

***Igf2,*** insulin like growth factor 2

***Ikzf1,*** IKAROS family zinc finger 1

***Il15,*** interleukin 15

***Il17ra,*** interleukin 17 receptor alpha

***Il18,*** interleukin 18

***Il1a,*** interleukin 1 alpha

***Il1b,*** interleukin 1 beta

***Il1r2,*** interleukin 1 receptor 2

***Il1rn,*** interleukin-1 receptor antagonist

***Il6,*** interleukin 6

***Irf5,*** interferon regulatory factor 5

***Irf8,*** interferon regulatory factor 8

***Isg15,*** ISG15 Ubiquitin Like Modifier

***Itga4,*** integrin subunit alpha 4

***Itgal,*** integrin subunit alpha L

***Itgax,*** integrin subunit alpha X

***Itgb2,*** integrin subunit beta 2

***Kif20a,*** kinesin family member 20a

***Laptm5,*** lysosomal protein transmembrane 5

***Lat2,*** linker for activation of T cells family member 2

***Lgals3,*** galectin 3

***Lipa,*** lipase A

***Loxl2,*** lysyl oxidase like 2

***Lpl,*** lipoprotein lipase

***Ltb,*** lymphotoxin beta

***Ltb4r2,*** leukotriene B4 receptor 2

***Mmp12,*** matrix metalloproteinase-12

***Mmp13,*** matrix metalloproteinase-13

***Mmp9,*** matrix metalloproteinase-9

***Mpeg1,*** macrophage expressed 1

***Ncf2,*** neutrophil cytosolic factor 2

***Nfatc2,*** nuclear factor of activated T cells 2

***Nlrp3,*** NLR family pyrin Domain Containing 3

***Nos2,*** nitric oxide synthase 2

***Olr1,*** oxidized low density lipoprotein receptor 1

***Pdgfa,*** platelet-derived growth factor subunit A

***Pdgfb,*** platelet-derived growth factor subunit B

***Pdgfra,*** platelet-derived growth factor receptor A

***Pdgfrb,*** platelet-derived growth factor receptor B

***Plau,*** plasminogen activator urokinase

***Pparg,*** peroxisome proliferator activated receptor gamma

***Psmb9,*** proteasome 20s subunit beta 9

***Ptafr,*** platelet activating factor receptor

***Ptprc,*** protein tyrosine phosphatase receptor type C

***Retnla,*** resistin like alpha

***Rgs1,*** regulator of G protein signaling 1

***Selplg,*** selectin P ligand

***Siglecf,*** sialic acid binding Ig like lectin f

***Sirpa,*** signal regulatory protein alpha

***Stat3,*** signal transducer and activator of transcription 3

***Syk,*** spleen associated tyrosine kinase

***Tgfb1,*** transforming growth factor beta 1

***Tlr13,*** toll-like receptor 13

***Tlr2,*** toll-like receptor 2

***Tlr4,*** toll-like receptor 4

***Tlr6,*** toll-like receptor 6

***Tlr7,*** toll-like receptor 7

***Tlr8,*** toll-like receptor 8

***Tlr9,*** toll-like receptor 9

***Tnf,*** tumor necrosis factor

***Tnfaip3,*** tnf alpha induced protein 3

***Tnfaip8,*** tnf alpha induced protein 8

***Tnfrsf11a,*** tnf superfamily member 11a

***Top2a,*** DNA topoisomerase II alpha

***Trem2,*** triggering receptor expressed on myeloid cells 2

***Tspan8,*** tetraspanin 8

***Tyrobp,*** tyrosine kinase-binding protein

***Usp18,*** ubiquitin specific peptidase 18

***Vav1,*** guanine nucleotide exchange factor 1

***Vcam1,*** vascular cell adhesion molecule 1

***Fgf2***

***Igf2***

***Nos2***

***Pdgfa***

***Pdgfb***

***Pdgfra***

***Pdgfrb***

***Tgfb1***

***Vcam1***

***Vegfa***

***Vegfc***

***Vegfa,*** vascular endothelial growth factor A

***Vegfc,*** vascular endothelial growth factor C

***Vsir,*** V-set immunoregulatory receptor

***Was,*** Wiskott-Aldrich syndrome actin nucleation promoting factor

***Acox1***

***Acad10***

***Acly***

***Adamts1***

***Elovl6***

***Fas***

***Hdac3***

***Hmgb1***

***Lipa***

***Pparg***

***Stat3***

1. **Supplementary Figure legends**

**Supplementary Figure 1. IFM-514 reduces macrophage infiltration in MCD-fed *ApoE^-/-^* mice.** F4/80 immunohistochemistry **(A)** and quantification **(B).** Results are expressed as the mean ± standard error of the mean (SEM); n=10/group, **p*<0.05 and ****p*<0.001. Representative photomicrographs were captured at 100× (scale bars=200μm) and 200× magnification (scale bars=100μm).

**Supplementary Figure 2. Serum interleukin levels and IFM-514 levels in WD-fed *ApoE^-/-^* mice after the IFM-514 treatment.** Groups (n=10) of 12-week-old *ApoE^-/-^* mice were fed *ad lib* for seven weeks with western diet (WD) diet as shown in the study design **(A)**. ). Concentration of IFM-514 in liver (ng/g of tissue) and serum (ng/mL) following i.p. administration of IFM-514 in NASH-induced mice for 20 consecutive days. The concentration was measured by HPLC-MS (**B-C**). Circulating IL-1β and IL-1α in IFM-514-treated WD-fed *ApoE^-/-^* mice (**D-E**). Results are expressed as mean ± standard error of the mean (SEM); n=10/group, *p<0.05, **p<0.01 and ***p<0.001. Abbreviations: IL1α, interleukin 1α.

**Supplementary Figure 3. IFM-514 effect in hepatic inflammation in WD-fed *ApoE^-/-^* mice.** H&E staining **(A)** inflammation score **(B)** and NAS score **(C).** F4/80 immunohistochemistry **(D),** F4/80 quantification **(E),** inflammasome-related gene expression **(F)**. Heatmap for hepatic inflammation set of genes **(G).** Results are expressed as the mean ± standard error of the mean (SEM); n=10/group, ^#^p<0.1. **p*<0.05, ***p*<0.01 and ****p*<0.001. Representative photomicrographs were captured at 100× (scale bars=200μm) and 200× magnification (scale bars=100μm). Abbreviations: Ccl1, chemokine (C-C motif) ligand 1; Ccl4, chemokine (C-C motif) ligand 4; Ccl28, chemokine (C-C motif) ligand 28; CV, central vein; H&E, hematoxylin and eosin; *Il-10*, interleukin 10; PV, portal vein. See the Table S4 for Heatmap genes.

**Supplementary Figure 4. IFM-514 effect in hepatic fibrosis and stellate cell activation in WD-fed *ApoE*^-/-^ mice**. Sirius red (SR) staining (**A**), α-smooth muscle actin immunostaining **(B),** SR quantification **(C)** α-smooth muscle actin quantification (**D**), hepatic 4-hydroxyproline content (**E)** and spleen pressure analyses **(F)** from WD-fed *ApoE^-/-^* mice. Heatmap for hepatic fibrosis (**G**) and portal hypertension (**H**) set of genes. All mRNA data were normalized to the expression of *18s*. Results are expressed as mean ± standard error of the mean (SEM); n=10/group, ^#^p<0.1, **p*<0.05, ***p*<0.01 and ****p*<0.001. Scale bars=200μm. Abbreviations: acta2, α-smooth muscle actin gene; αSMA, α-smooth muscle actin; col1a1, collagen type 1 α1; CV, central vein; PV, portal vein. See the Table S4 for Heatmap genes.
